# Effect of ENaC Modulators on Rat Neural Responses to NaCl

**DOI:** 10.1371/journal.pone.0098049

**Published:** 2014-05-19

**Authors:** Shobha Mummalaneni, Jie Qian, Tam-Hao T. Phan, Mee-Ra Rhyu, Gerard L. Heck, John A. DeSimone, Vijay Lyall

**Affiliations:** 1 Department of Physiology and Biophysics, Virginia Commonwealth University, Richmond, Virginia, United States of America; 2 Korea Food Research Institute, Bundang-gu, Sungnam-si, Gyeonggi-do, Korea; Duke University Medical Center, United States of America

## Abstract

The effects of small molecule ENaC activators N,N,N-trimethyl-2-((4-methyl-2-((4-methyl-1H-indol-3-yl)thio)pentanoyl)oxy)ethanaminium iodide (Compound 1) and N-(2-hydroxyethyl)-4-methyl-2-((4-methyl-1H-indol-3-yl)thio)pentanamide (Compound 2), were tested on the benzamil (Bz)-sensitive NaCl chorda tympani (CT) taste nerve response under open-circuit conditions and under ±60 mV applied lingual voltage-clamp, and compared with the effects of known physiological activators (8-CPT-cAMP, BAPTA-AM, and alkaline pH), and an inhibitor (ionomycin+Ca^2+^) of ENaC. The NaCl CT response was enhanced at −60 mV and suppressed at +60 mV. In every case the CT response (*r*) versus voltage (*V*) curve was linear. All ENaC activators increased the open-circuit response (*r_o_*) and the voltage sensitivity (*κ*, negative of the slope of the *r* versus *V* curve) and ionomycin+Ca^2+^ decreased *r_o_* and *κ* to zero. Compound 1 and Compound 2 expressed a sigmoidal-saturating function of concentration (0.25–1 mM) with a half-maximal response concentration (*k*) of 0.49 and 1.05 mM, respectively. Following treatment with 1 mM Compound 1, 8-CPT-cAMP, BAPTA-AM and pH 10.3, the Bz-sensitive NaCl CT response to 100 mM NaCl was enhanced and was equivalent to the Bz-sensitive CT response to 300 mM NaCl. Plots of *κ* versus *r_o_* in the absence and presence of the activators or the inhibitor were linear, suggesting that changes in the affinity of Na^+^ for ENaC under different conditions are fully compensated by changes in the apical membrane potential difference, and that the observed changes in the Bz-sensitive NaCl CT response arise exclusively from changes in the maximum CT response (*r_m_*). The results further suggest that the agonists enhance and ionomycin+Ca^2+^ decreases ENaC function by increasing or decreasing the rate of release of Na^+^ from its ENaC binding site to the receptor cell cytosol, respectively. Irrespective of agonist type, the Bz-sensitive NaCl CT response demonstrated a maximum response enhancement limit of about 75% over control value.

## Introduction

In rats, about 70% of the chorda tympani (CT) taste nerve response to NaCl is due to Na^+^ influx through the amiloride- and benzamil (Bz)-sensitive epithelial Na^+^ channel (ENaC) expressed in the apical membrane of a subset of fungiform taste bud cells, and is associated with appetitive behavioral responses to low NaCl concentrations [1]–[4]. In contrast, high salt concentrations are aversive, and seem to recruit the two primary aversive taste pathways by activating sour- and bitter-sensing taste bud cells [4]. ENaC is a heterotrimeric constitutively active Na^+^ channel composed of α, β and γ subunits. In addition to α, β and γ subunits, human taste cells express the δ hENaC subunit [5]. Similar to α hENaC, δ hENaC can form functional amiloride-sensitive channels when expressed alone or in combination with βγ hENaC. In rats, inhibiting ENaC activity with amiloride appears to render NaCl qualitatively indistinguishable from KCl [6]. In mice, selectively silencing α-ENaC activity in taste cells abolishes the appetitive taste of NaCl [3]. In contrast, no significant effect of amiloride is observed on perceived salt taste intensity in human subjects even when tested at levels at approximately 300-fold above the IC_50_ for αβγ ENaC expressed in oocytes and equivalent to approximately 10-fold over the IC_50_ value for δβγ ENaC expressed in oocytes [7]–[9]. These differences reflect not only the inherently different properties of hENaC and rENaC or mENaC but also their relative contributions to salt taste sensing in humans and rodent models, respectively.

Recently, a small molecule activator, N-(2-hydroxyethyl)-4-methyl-2-((4-methyl-1H-indol-3-yl)thio)pentanamide of hENaC has been described. It demonstrated a threshold for activating hENaC expressed in frog oocytes at around 30 nM and produced a half-maximal response at 1.2 µM. It produced around 700% increase in the amiloride-sensitive Na^+^ current above baseline with a Hill coefficient of 1.0 [7]. In another study [10], between 0.03 and 10 µM, N-(2-hydroxyethyl)-4-methyl-2-((4-methyl-1H-indol-3-yl)thio)pentanamide depolarized the membrane potential in hENaC expressing cells in a concentration-dependent manner with an EC_50_ value of 1.26±0.29 µM, and Bz effectively inhibited its action. In contrast, it did not activate αβγ mENaC unless used at 100–300 µM, more than 2 log orders above concentrations required to induce half-maximal hENaC activation [7]. However, at present the effect of N-(2-hydroxyethyl)-4-methyl-2-((4-methyl-1H-indol-3-yl)thio)pentanamide and related small molecule activators of ENaC on the amiloride- and benzamil (Bz)-sensitive NaCl CT responses in rodent models have not been investigated. Specifically, the concentration range over which small molecule ENaC enhancers increase the NaCl CT response and the extent to which the maximum CT response can be increased at high enhancer concentration are as yet undetermined. In addition, it is not known if the magnitude of the enhancement in the NaCl CT response in the presence of ENaC enhancers is comparable to or exceeds that observed in the presence of the physiological modulators of taste cell ENaC, such as an increase in intracellular pH (pH_i_) [11], a decrease in intracellular Ca^2+^ ([Ca^2+^]_i_) [12], a decrease in cell volume [13], and an increase in 3′-5′-cyclic adenosine monophosphate (cAMP) [14]. Accordingly, the aims of this study were first, to investigate if the low molecular weight structurally related activators of ENaC, N,N,N-trimethyl-2-((4-methyl-2-((4-methyl-1H-indol-3-yl)thio)pentanoyl)oxy) ethanaminium iodide (Compound 1) and N-(2-hydroxyethyl)-4-methyl-2-((4-methyl-1H-indol-3-yl)thio)pentanamide (Compound 2) [7], modulate rat Bz-sensitive NaCl CT responses under open-circuit conditions and at ±60 mV applied lingual potential, and second, to determine if the effects of Compound 1 and Compound 2 are comparable to the enhancement in the Bz-sensitive NaCl response observed by decreasing taste cell [Ca^2+^]_i_ with BAPTA-AM [12], increasing taste cell cAMP levels by topical lingual application of the membrane permeable 8-chlorophenylthio (CPT)-cAMP [14], and increasing taste cell pH_i_ by increasing the NaCl stimulating solution pH (pH_o_) from 7.0 to 10.3 [11]. In addition, CT responses were also monitored under conditions where taste cell [Ca^2+^]_i_ was increased using ionomycin +10 mM CaCl_2_ which inhibits the Bz-sensitive NaCl CT response to baseline [12]. The CT responses were monitored under open-circuit conditions and under lingual voltage clamp conditions to permit us to analyze the data using a kinetic model of the CT response that assumes that the response is proportional to the Na^+^ flux through ENaC [15].

Our results show that Compound 1 and Compound 2 increased the open-circuit Bz-sensitive NaCl CT response (*r_o_*) in a concentration-dependent manner with a half-maximal response concentration (*k*) of 0.49 mM and 1.05 mM, respectively. Both agonists increased the voltage sensitivity (*κ*) of the NaCl CT response and *r_o_*, consistent with an increase in the apical membrane Na^+^ conductance of ENaC expressing fungiform taste bud cells. Similarly, an increase in taste cell pH_i_ and cAMP and a decrease in [Ca^2+^]_i_ caused an increase in both *κ* and *r_o_*. In contrast, an increase in [Ca^2+^]_i_ decreased both *κ* and *r_o_*. A plot of *κ* against *r_o_* in the absence and presence of ENaC activators and inhibitor was linear. The model suggests that the above ENaC modulators exert their effects by either enhancing or inhibiting the maximum Bz-sensitive NaCl CT response (*r_m_*) and by altering the rate constant (*k_c_*) for the dissociation of Na^+^ from the channel into the cell interior. In contrast to the studies on hENaC expressed in oocytes [7], the maximum enhancement (*r_m_*) in the Bz-sensitive NaCl CT response in the intact rat sensory system in the presence of the above ENaC modulators was around 75%.

## Materials and Methods

N,N,N-trimethyl-2-((4-methyl-2-((4-methyl-1H-indol-3-yl)thio)pentanoyl)oxy) ethanaminium iodide (Compound 1) and N-(2-hydroxyethyl)-4-methyl-2-((4-methyl-1H-indol-3-yl)thio)pentanamide (Compound 2) were obtained from Senomyx Inc., San Diego, CA. 8-(4-Chlorophenylthio) adenosine 3′,5′-cyclic monophosphate (8-CPT-cAMP), 8-(4- Chlorophenylthio)adenosine- 3′,5′- cyclic monophosphorothioate, Rp-isomer (Rp-8-CPT-cAMPS), 8-(4- Chlorophenylthio) guanosine- 3′,5′- cyclic monophosphorothioate (8-CPT-cGMP); 3-Isobutyl-1-methylxanthine (IBMX), forskolin, benzamil (Bz), 1,2-Bis(2-aminophenoxy)ethane-N,N,N′,N′-tetraacetic acid tetrakis(acetoxymethyl ester) (BAPTA-AM), 4-(2-Hydroxyethyl)piperazine-1-ethanesulfonic acid (HEPES), tris(hydroxymethyl)aminomethane (TRIS) and N-methyl -D-glucamine hydrochloride (NMGD-Cl) were obtained from Sigma-Aldrich.

### CT Taste Nerve Recordings

The animals were housed in the Virginia Commonwealth University (VCU) animal facility in accordance with institutional guidelines. All *in vivo* and *in vitro* animal protocols were approved by the Institutional Animal Care and Use Committee of VCU. Thirty six female Sprague-Dawley (SD) rats (120–150 g; obtained from Charles River Laboratory, Raleigh, NC) were anesthetized by intraperitoneal injection of pentobarbital (60 mg/Kg) and supplemental pentobarbital (20 mg/Kg) was administered as necessary to maintain surgical anesthesia. The animal’s corneal reflex and toe-pinch reflex were used to monitor the depth of surgical anesthesia. Body temperatures were maintained at 36–37° with an isothermal pad (Braintree Scientific, Braintree MA). The left CT nerve was exposed laterally as it exited the tympanic bulla and placed onto a 32G platinum/iridium wire electrode. An indifferent electrode was placed in nearby tissue. Stimulus solutions maintained at room temperature were injected into a Lucite chamber affixed by vacuum to a 28 mm^2^ patch of anterior dorsal lingual surface. The chamber was fitted with separate Ag-AgCl electrodes for measurement of current and potential and served as inputs to a voltage-current clamp amplifier that permitted the recording of CT responses with the chemically stimulated receptive field under zero current-clamp or voltage-clamp. The potentials were referenced to the mucosal side of the tongue and clamp voltages were measured relative to the open circuit potential [16]. For stimulation or rinsing, 3-ml aliquots were injected at a rate of 1 ml/s into the perfusion chamber. Neural responses were differentially amplified with a custom built, optically-coupled isolation amplifier. For display, responses were filtered using a band pass filter with cutoff frequencies 40 Hz–3 KHz and fed to an oscilloscope. Responses were then full-wave rectified and integrated with a time constant of 1 s. Integrated responses were typically taken for 1–2 min and were quantified by calculating the mean over the final 30 s of the response. Mean responses were then normalized by dividing them by the mean response to 300 mM NH_4_Cl over a similar final 30 s period. The normalized data were reported as the mean ± standard error of the mean (SEM) of the number of animals. Responses to control stimuli consisting of 300 mM NH_4_Cl applied at the beginning and at the end of experiment were used to assess preparation stability. The preparation was considered stable only if the difference between the magnitude of the control stimuli at the beginning and at the end of the experiment was less than 10% [17]. Integrated neural responses and lingual current and voltage changes were captured on disk using LabView software (National Instruments, Austin, TX) and analyzed off-line as described previously [18].

#### Data analysis

The dependence of the Bz-sensitive part of the NaCl CT response on the concentration of agonists was fitted to a modified Hill Equation of the form:
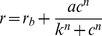
(1)


Here, *r* is the normalized Bz-sensitive part of the CT response, *r_b_* is the response when the concentration of the agonist, *c*, is zero, *a* is the response level above *r_b_* achieved as *c* becomes large, *k* is the concentration of the agonist at which *r* = *r_b_* +0.5*a*, and *n* is a number greater than one [19]. The data points representing changes in the total NaCl CT response, the Bz-sensitive part of the NaCl response, or the Bz-insensitive part of the NaCl response with applied lingual voltage were fitted to least square lines and further analyzed according a kinetic model of ion channel activity [15], [18]. All fitting parameters under different experimental conditions are reported in the figure legends or in Tables.

### Taste Stimuli

Table 1 lists the different rinse and NaCl solutions used to stimulate the rat tongue during CT recordings under open-circuit conditions and under ±60 mV lingual voltage clamp. Compound 1 and Compound 2-induced changes in the NaCl CT responses were compared with changes in the NaCl CT responses produced by altering taste cell pH (pH_i_), Ca^2+^ ([Ca^2+^]_i_), and cAMP [11], [12], [14]. Table 1 also lists the different solutions containing specific activators and blockers that were applied to the tongue topically to effect changes in fungiform taste cell pH_i_, [Ca^2+^]_i_, cAMP, cGMP and protein kinase A (PKA). Direct measurement on isolated polarized fungiform taste bud cells showed that pH_i_ changes can be altered by varying the apical solution pH (pH_o_), such that, a given change in pH_o_ corresponded to a unique change in pH_i_ [20]. In previous studies, the maximum increase in the Bz-sensitive NaCl CT response was obtained by stimulating the tongue with 100 mM NaCl solutions at pH 10.3 relative to pH 7.0 [11]. Taste cell [Ca^2+^]_i_ can be decreased *in vivo* by exposing the lingual epithelium to membrane-permeable BAPTA-AM. In previous studies [12], topical lingual application of 33 mM BAPTA-AM for 30 min produced the maximum increase in the Bz-sensitive NaCl CT response. Taste cell [Ca^2+^]_i_ can be increased by exposing the tongue to ionomycin in the presence of extracellular Ca^2+^. In previous studies [12], topical lingual application of 150 µM ionomycin +10 mM CaCl_2_ for 30 min inhibited the Bz-sensitive NaCl CT response to the rinse baseline level.

**Table 1:** Composition of solutions used in CT experiments.

	Stimulating solutions (mM)	Rinse (mM)
	100 NaCl	10 KCl
	100 NaCl +0.005 Bz	10 KCl
	100 NaCl +0.25 to 1.0 Compound 1	10 KCl
	100 NaCl +0.005 Bz +0.25–1.0 Compound 1	10 KCl
	100 NaCl +0.25 to 1.0 Compound 2	10 KCl
	100 NaCl +0.005 Bz +0.25–1.0 Compound 2	10 KCl
	100 NaCl +10 HEPES (pH 7.0)	10 KCl +10 HEPES (pH 7.0)
	100 NaCl +10 HEPES +0.005 Bz (pH 7.0)	10 KCl +10 HEPES (pH 7.0)
	100 NaCl +10 TRIS (pH 10.3)	10 KCl +10 TRIS (pH 10.3)
	100 NaCl +10 TRIS +0.005 Bz (pH 10.3)	10 KCl +10 TRIS (pH 10.3)
	50–1000 NaCl	10 KCl
	50–1000 NaCl +0.005 Bz	10 KCl
	300 NH_4_Cl	10 KCl
**Taste cell**	**Topical lingual application (mM)**	**Time (min)**
↓ [Ca^2+^]_i_	33 BAPTA-AM	30
↑ [Ca^2+^]_i_	0.15 ionomycin +10 CaCl_2_	30
↑ cAMP	20 8-CPT-cAMP	30
↑ cAMP	0.1 IBMX +0.1 forskolin	20
↑ cGMP	20 8-CPT-cGMP	30
↓ PKA	4 Rp-8-CPT-cAMP	20
↑ pH_i_	10 HEPES (pH 7.0) to 10 TRIS (pH 10.3)	

CT responses were monitored under open-circuit conditions and under ±60 mV lingual voltage clamp.

pH of the solutions containing HEPES or TRIS was adjusted with HCl or NaOH.

In preliminary studies, 8-CPT-cAMP was applied topically to the anterior lingual surface of rat tongues at varying concentrations ranging between 5 and 20 mM and for different time intervals between 0 and 30 min. Application of 8-CPT-cAMP produced a dose-dependent increase in the NaCl CT response producing the maximum increase in the response between 15 and 20 mM (data not shown). The effects of 8-CPT-cAMP could only be observed after 10–15 min of its lingual application (data not shown). Accordingly, here we monitored the NaCl CT response before and after topical lingual application of 20 mM 8-CPT-cAMP for 30 min. In some studies rat tongue was pretreated with Rp-8-CPT-cAMP before applying 8-CPT-cAMP. Rp-8-CPT-cAMP is an inhibitor of the activation by cAMP of cAMP-dependent protein kinase I and II [21]. In additional experiments endogenous cAMP levels were increased in fungiform taste bud cells by treating the tongue with IBMX+forskolin. Forskolin activates adenylate cyclase to facilitate the conversion of adenosine 5′-triphosphate (ATP) to cAMP [22], and IBMX inhibits the conversion of cAMP to adenosine monophosphate (AMP) by phosphodiesterases [23]. As a control we also investigated the effect of topical lingual application of 8-CPT-cGMP on the NaCl CT response (Table 1).

### Na^+^ Imaging

Four rats were anesthetized by exposing them to an inhalation anesthetic, isoflurane (1.5 ml) in a desiccator. When rats were fully unconscious, a midline incision was made in the chest wall and the aorta severed. The tongues were then rapidly removed and stored in ice-cold control Ringer’s solution (Table 2). The lingual epithelium was isolated by collagenase treatment. A small piece of the anterior lingual epithelium containing a single fungiform papilla was mounted in a special microscopy chamber as described earlier [24].

**Table 2:** Composition of Ringer’s solutions (mM).

Salt	Control Ringer’s	150 Na^+^- Ringer’s	a0 Na^+^-Ringer’s
NaCl	140	150	0
KCl	5	5	5
CaCl_2_	1	1	1
MgCl_2_	1	1	1
Na-pyruvate	10	0	0
Glucose	10	10	10
HEPES	10	10	10
NMDG-Cl	0	0	150
pH	7.4	7.4	7.4

a In some experiments the 0 Na^+^ Ringer’s solution contained in addition, 150 µM 8-CPT-cAMP or 3 µM ionomycin.

The pH of the solutions was adjusted with HCl or NaOH.

For the measurement of unilateral apical Na^+^ influx, taste cells within the taste bud were loaded with sodium green (10 µM) dissolved in control Ringer’s solution (Table 2) for 2 h. After loading the tissue was initially perfused on both sides with 0 Na^+^ Ringer’s solution (Table 2). The change in F_490_ was measured as a response to a unilateral switch in the apical compartment from 0 Na^+^ Ringer’s solution to control Ringer’s solution before and after 15 min exposure of the basolateral membrane of taste bud cells to 250 µM 8-CPT-cAMP. Small regions of interest (ROIs) in the taste bud (diameter 2–3 µm) were chosen in which the changes in the fluorescence intensity (F_490_) were analyzed using imaging software (TILLvisIon v 4.0.7.2; TILL Photonics, Martinsried, Germany). Each ROI contained 2–3 taste cells. Thus the fluorescence intensity recorded for a ROI represents the mean value from 2–3 taste cells within the ROI. In a typical experiment the FIR measurements were made in an optical plane in the taste bud containing 6 ROIs (approximately 12–18 cells). The background and auto-fluorescence at 490 nm were corrected from images of a taste bud without the dye [11].

#### Data analysis

In taste bud cells loaded with Na-green the changes in Na^+^ were expressed relative to the fluorescence intensity (F_490_) under control conditions. The F_490_ under control conditions for each ROI was taken as 100%. Student’s t-test was employed to analyze the differences between sets of data [12].

## Results

### Effect of Compound 1 on the Open-circuit NaCl CT Response

**Figure 1:**
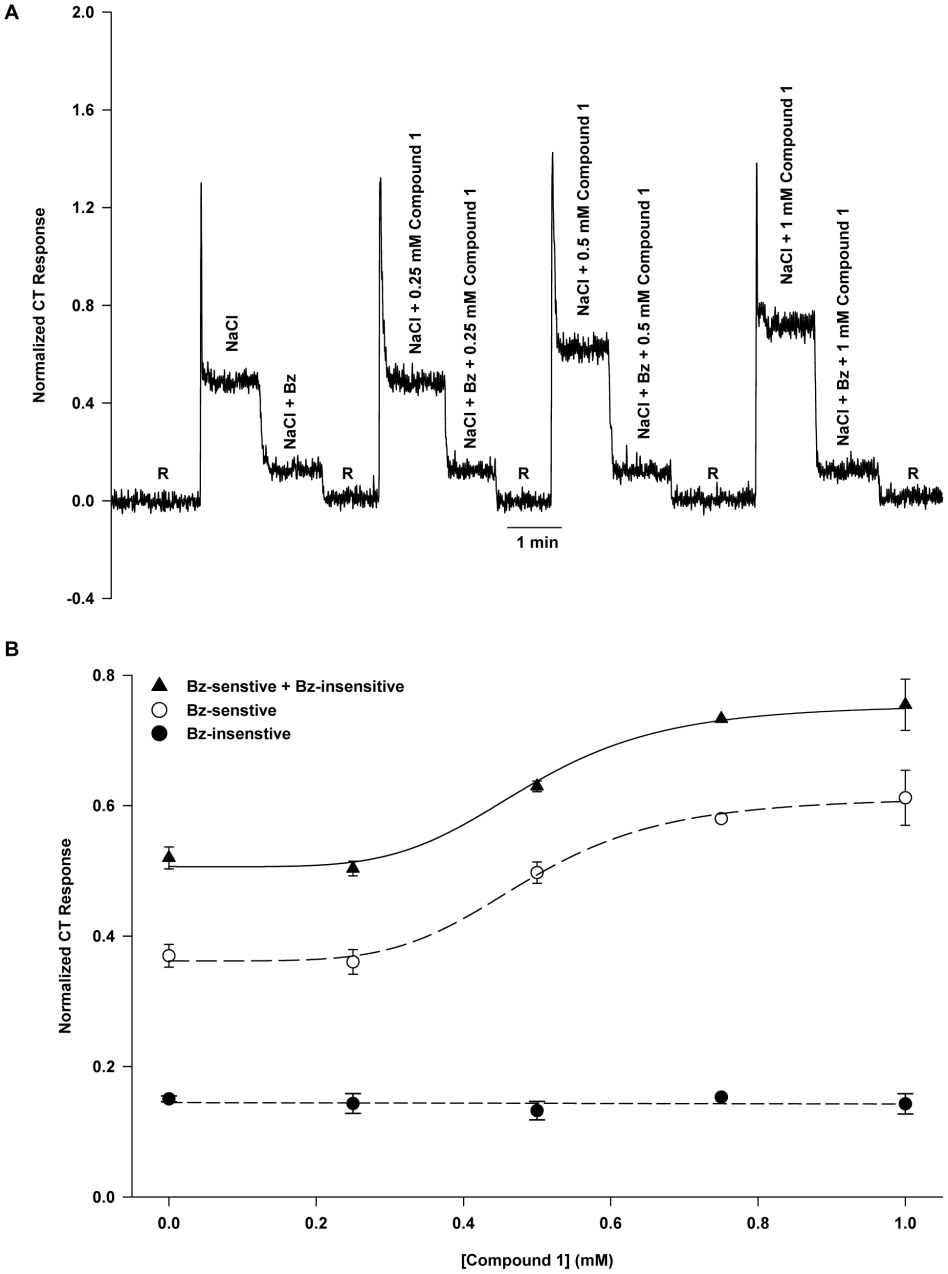
Effect of Compound 1 on rat NaCl CT response. (**A**) Representative CT responses to 100 mM NaCl containing either 0, 0.25 mM, 0.5 mM or 1 mM Compound 1 in the presence and absence of 5 µM Bz relative to 10 mM KCl rinse (R). (**B**) The Bz-insensitive part of the NaCl response (•) does not vary with Compound 1 concentration. The regression line has intercept = 0.145 and slope = −0.0021. The latter does not differ from zero, so the Bz-insensitive response is 0.145 for any Compound 1 concentration. (Compound 1 = 0 mM, n = 6; Compound 1 = 0.25, 0.5, and 1 mM, n = 3; Compound 1 = 0.75 mM, n = 1; n = number of animals). The mean Bz-sensitive part of the NaCl CT response (○) is the mean total response minus the mean Bz-insensitive part of the response. The curve is fitted according to eq. 1. The equation parameters are: *r_b_* = 0.362, *a* = 0.251, *k* = 0.490 mM, and *n* = 5.2. The n values are same as in Fig. 1B. The data points (▴) represent the mean total response to 100 mM NaCl at 0, 0.25 mM, 0.5 mM, 0.75 mM, or 1 mM Compound 1. The curve is the sum of the regression curves for the Bz-insensitive part of the response (•) and the Bz-sensitive part of the NaCl CT response (○). The values are mean ± SEM of number of animals (n).

Figure 1A shows the typical effect of increasing Compound 1 concentrations on the rat open-circuit NaCl CT response relative to the 10 mM KCl rinse (R). The main effect of Compound 1 was on the enhancement of the tonic NaCl CT response. No enhancement was observed at 0.25 mM Compound 1, however, at 0.5 mM and 1 mM, Compound 1 caused the NaCl CT response to increase. Compound 1 had no effect on the CT response to NaCl+Bz. Thus, only the Bz-sensitive component ((NaCl response)-(NaCl+Bz response)) of the NaCl CT response was affected. Figure 1B (•) shows the mean Bz-insensitive part of the NaCl response. The data points were fitted to a regression line. The slope (−0.0021±0.012) was not statistically different from zero (*P* = 0.87), confirming that the Bz-insensitive part of the response was not affected by Compound 1. The effect of Compound 1 on the Bz-sensitive part of the NaCl response is shown in Fig. 1B (○). The Bz-sensitive part of response increased as a sigmoidal saturating function of Compound 1 concentration. The data points were fitted using least squares criteria, to eq 1. The value of *n* is a positive number, suggesting that Compound 1 acts through a cooperative mechanism. The maximum possible response for large Compound 1 concentration is, therefore, *r_b_*+*a* = 0.613, so Compound 1 increases *r* by a maximum of 70%. The whole CT response is the sum of its Bz-insensitive and the Bz-sensitive parts. Figure 1B (▴) shows the variation of the whole CT response to NaCl as a function of the Compound 1 concentration. The fitted curve is, as expected, just the sum of the regression line fitting the Bz-insensitive part (•) and the sigmoidal-saturating curve fitting the Bz-sensitive part (○ and eq. 1).

### Effect of Compound 1 on NaCl CT Response Under Voltage-clamp

**Figure 2:**
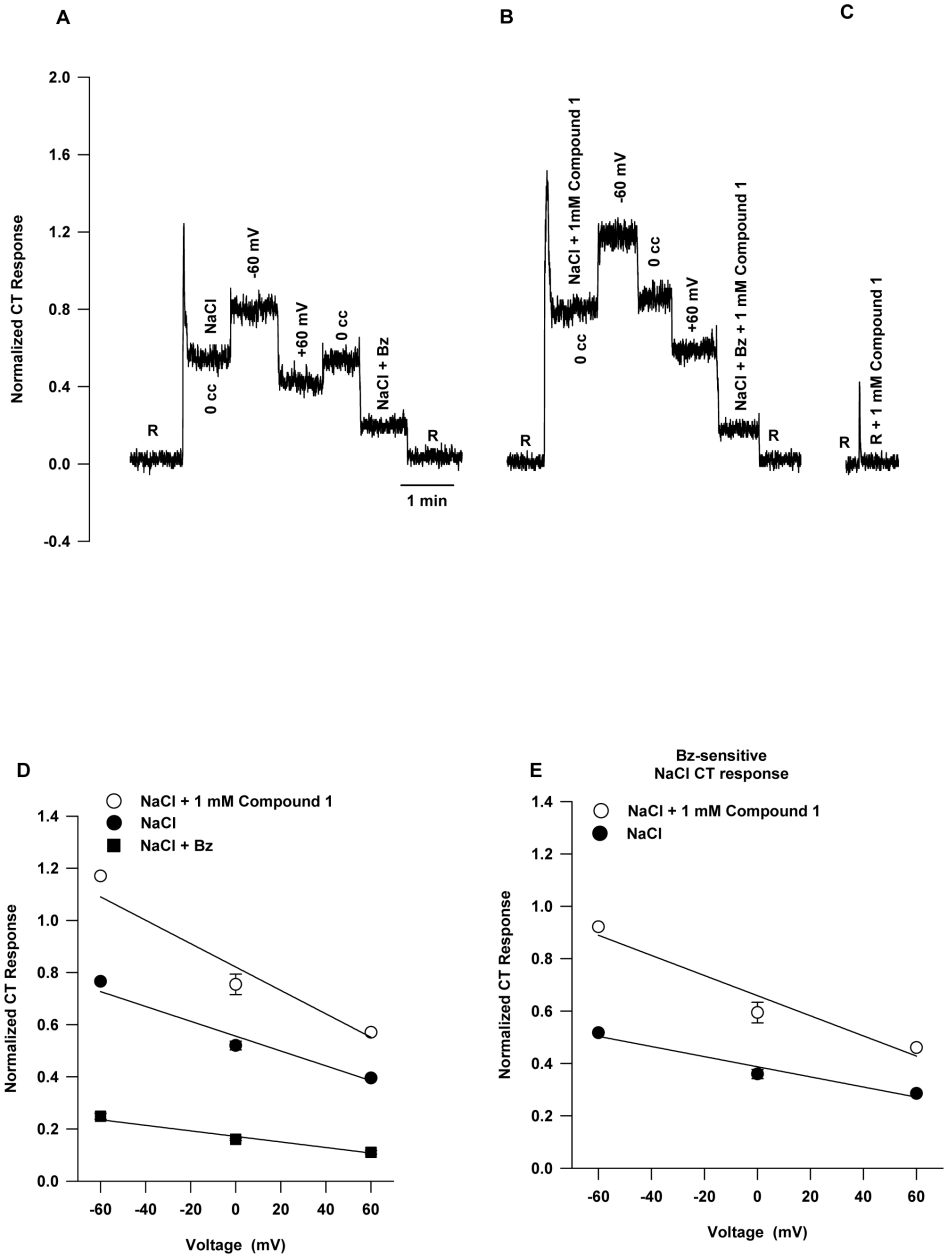
Effect of lingual voltage clamp on rat NaCl CT response in the absence and presence of Compound 1. (**A**) A representative CT response to 100 mM NaCl under open-circuit (0 cc), and at −60 mV and +60 mV and NaCl+Bz response under open-circuit relative to 10 mM KCl rinse (R). (**B**) Response to 100 mM NaCl +1 mM Compound 1 under open-circuit (0 cc), and at −60 mV and +60 mV and NaCl+Bz+Compound 1 response under open-circuit. (**C**) Compound 1 (1 mM) added to the rinse (R) gave no tonic CT response. (**D**) NaCl+Bz gives the smallest open-circuit response, *r_o_*, (0.173±0.009; response at *V* = 0) and lowest voltage sensitivity (0.0011±0.0002; negative of the slope of the *r* versus *V* line, *κ*). The NaCl curve represents the sum of the Bz-sensitive and the Bz-insensitive responses without Compound 1 (*r_o_* = 0.561±0.029; *κ* = 0.0031±0.0006) and the NaCl+Compound 1 curve is the sum of the Bz-sensitive and the Bz-insensitive responses in the presence of Compound 1 (*r_o_* = 0.832±0.055; *κ* = 0.0050±0.0011). For the NaCl line the increase in *r_o_* represents the presence of ENaC and the increased voltage sensitivity indicates the increase in taste cell membrane conductance due to ENaC. The NaCl+Compound 1 line shows the effect of pharmacologically increasing ENaC conductance (greater increase in open-circuit response and voltage sensitivity). (**E**) The Bz-sensitive part of the NaCl CT response with and without 1 mM Compound 1. Compound 1 increases both the voltage sensitivity (*κ*) and the open-circuit response (*r_o_*) consistent with modulation of ENaC activity exclusively (cf. Fig. 1A and 1B). The values are mean ± SEM of 3 rats.

Figure 2A shows a response to NaCl under open-circuit, at −60 mV and +60 mV lingual voltage clamp, and under open-circuit with added Bz. Relative to open-circuit, −60 mV lingual voltage clamp enhanced the response while +60 mV lingual voltage clamp suppressed the response. The effect of the presence of 1 mM Compound 1 added to each stimulus solution is seen in Fig. 2B. Relative to control responses (Fig. 2A), responses under open-circuit were enhanced as was the size of the difference in responses between −60 mV and +60 mV (see also Fig. 2D). Relative to control conditions, adding 1 mM Compound 1 to NaCl+Bz had no effect on the open-circuit response (cf. Figs. 1A and 1B). Fig. 2C shows that 1 mM Compound 1 dissolved in the rinse solution (R) when applied to the tongue produced no tonic CT response of its own.

Figure 2D shows that the response was a linear function of voltage for NaCl+Bz (Bz-insensitive response), NaCl, and NaCl +1 mM Compound 1. Each line is characterized by its voltage sensitivity, *κ*, (negative of the slope) and open-circuit response, *r_o_* (response axis intercept at *V* = 0). As shown below, *κ* is related to the global conductance of an apical membrane taste transducer ion channel and is proportional to *r_o_*. For the NaCl+Bz line, *κ* represents the voltage sensitivity that derives from the Na^+^ flux through all the Bz-insensitive cation channel transducers contributing to the CT response and *r_o_* is the corresponding Bz-insensitive open-circuit response [18]. Since these transducers always produce a response that is smaller than that produced by the sum of all the ENaC transducers, for the NaCl+Bz line, *r_o_* and *κ* have the smallest values in the set of three curves in Fig. 2D.

Since Compound 1 modulates the Bz-sensitive part of the response exclusively (Fig. 1B) it is useful to isolate this part of the response for further analysis (see below). The Bz-sensitive part of the response was found as the difference between the total NaCl response and the Bz-insensitive part of the response. Fig. 2E illustrates the increase in *r_o_* and *κ* due to Compound 1 in the Bz-sensitive part of the NaCl CT response. The values of the parameters *κ* and *r_o_* for the Bz-sensitive response line without and with Compound 1 are shown in Table 3.

**Table 3:** Normalized values of the voltage-sensitivity (*κ*) and the open-circuit Bz-sensitive NaCl CT response (*r_o_*) in the absence and presence of ENaC modulators.

ENaC Modulator	*κ*	*r_o_*
**Control**	0.0019±0.0004	0.388±0.020
**Compound 1**	0.0038±0.0009	0.659±0.046
**Control**	0.0019±0.0005	0.390±0.020
**Compound 2**	0.0032±0.0004	0.527±0.019
**Control**	0.0016±0.0004	0.280±0.018
**8-CPT-cAMP**	0.0037±0.0008	0.629±0.038
**Control**	0.0021±0.0001	0.474±0.005
**BAPTA-AM**	0.0036±0.0002	0.699±0.008
**Control (pH_o_ 7.0)**	0.0018±0.0003	0.373±0.017
**pH_o_ 10.3**	0.0040±0.0005	0.611±0.027
**Control**	0.0022±0.0004	0.318±0.002
**Ionomycin+CaCl_2_**	−0.0001±0.0001	−0.009±0.004
**Mean control value**	0.0019±0.0001	0.370±0.027

Values are mean ± SEM of 3 animals in each group.

The Mean control value represents the mean of all control parameters under the 6 different experimental conditions.

### Effect of Compound 2 on the Open-circuit NaCl CT Response

**Figure 3:**
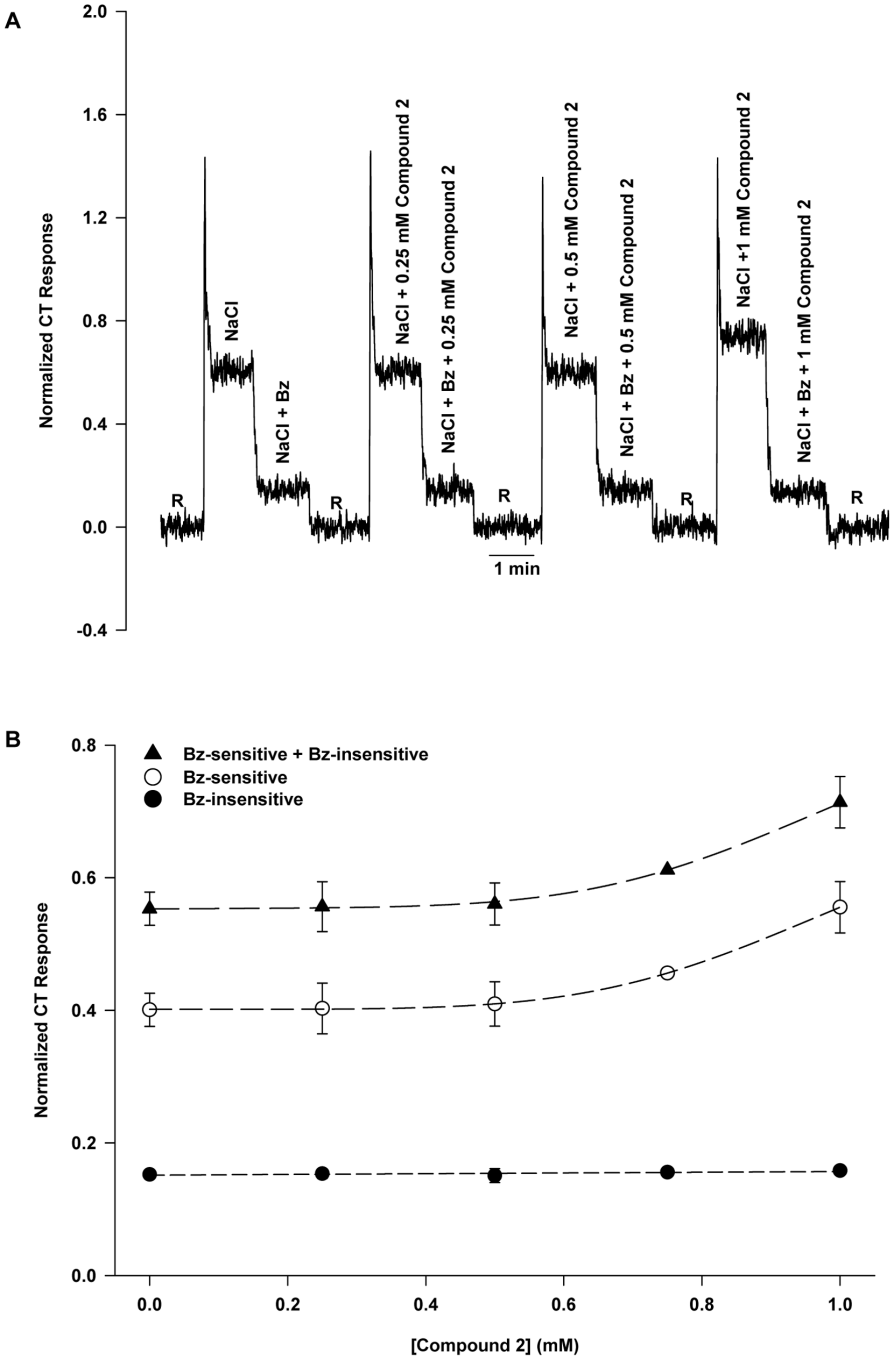
Effect of Compound 2 on rat NaCl CT response. (**A**) Representative CT responses to 100 mM NaCl containing either 0, 0.25 mM, 0.5 mM or 1 mM Compound 2 in the presence and absence of 5 µM Bz relative to 10 mM KCl rinse (R). Responses to NaCl+Bz are unaffected by Compound 2. (**B**) Similar to Fig. 1B, the Bz-insensitive part of the NaCl CT response (•) does not vary with Compound 2 concentration. The regression line has intercept = 0.151 and slope = 0.005. The latter does not differ from zero, so the Bz-insensitive response is 0.151 for any Compound 2 concentration. (Compound 2 = 0 mM, n = 6; Compound 2 = 0.25, 0.5, and 1 mM, n = 3; Compound 2 = 0.75 mM, n = 1; n = number of animals). The mean Bz-sensitive part of the NaCl CT response to Compound 2 (○) was fitted according to eq. 1. The equation parameters are: *r_b_* = 0.402, *a* = 0.348, *k* = 1.05 mM, and *n* = 5.0. Similar to Fig. 1B, the data points (▴) represent the mean total response to 100 mM NaCl at 0, 0.25 mM, 0.5 mM, 0.75 mM, or 1 mM Compound 2. The curve is the sum of the regression curves for the Bz-insensitive part of the response (•) and the Bz-sensitive part of the NaCl CT response (○). The values are mean ± SEM of number of animals (n).

Figure 3A shows that the pattern of the effect of increasing the Compound 2 concentration on the open-circuit response was similar to that of Compound 1 (cf. Fig. 1A). However, unlike Compound 1 enhancement was not observed at 0.5 mM, but it was evident at 1 mM Compound 2. Similar to Compound 1, Compound 2 affected only the Bz-sensitive component of the response. Figure 3B (•) shows the mean Bz-insensitive part of the NaCl response. The data points were fitted to a regression line. The slope (0.0056±0.0029) was not statistically different from zero (*P* = 0.15), confirming that like Compound 1, the Bz-insensitive part of the response was not affected by Compound 2. Fig. 3B (○) shows the Bz-sensitive part of the response was unaffected by Compound 2 at concentrations of 0.5 mM and less. However, 0.75 mM and 1 mM Compound 2 show a rising response. Equation 1 was used to fit the data points (○) on the assumption that, like the enhanced response due to Compound 1, the enhanced response due to Compound 2 will also saturate at concentrations above 1 mM. The positive slope of the curve at a Compound 2 concentration of 1 mM suggests the response will continue to increase before saturating. The predicted maximum response for large Compound 2 concentration is, therefore, *r_b_* (0.402)+*a* (0.348) = 0.750, or *r* is predicted to increase due to Compound 2 by a maximum of 87%. Like Compound 1, the effect of Compound 2 on the whole NaCl response (Fig. 3B; ▴) can be represented as the sum of the Bz-insensitive regression line (•) and the Bz-sensitive curve (○). Taken together, the above results show that both Compound 1 and Compound 2 are effective enhancers of the Bz-sensitive part of the NaCl response, but that Compound 1 has a lower enhancer threshold.

### Effect of Compound 2 on NaCl CT Response Under Voltage-clamp

**Figure 4:**
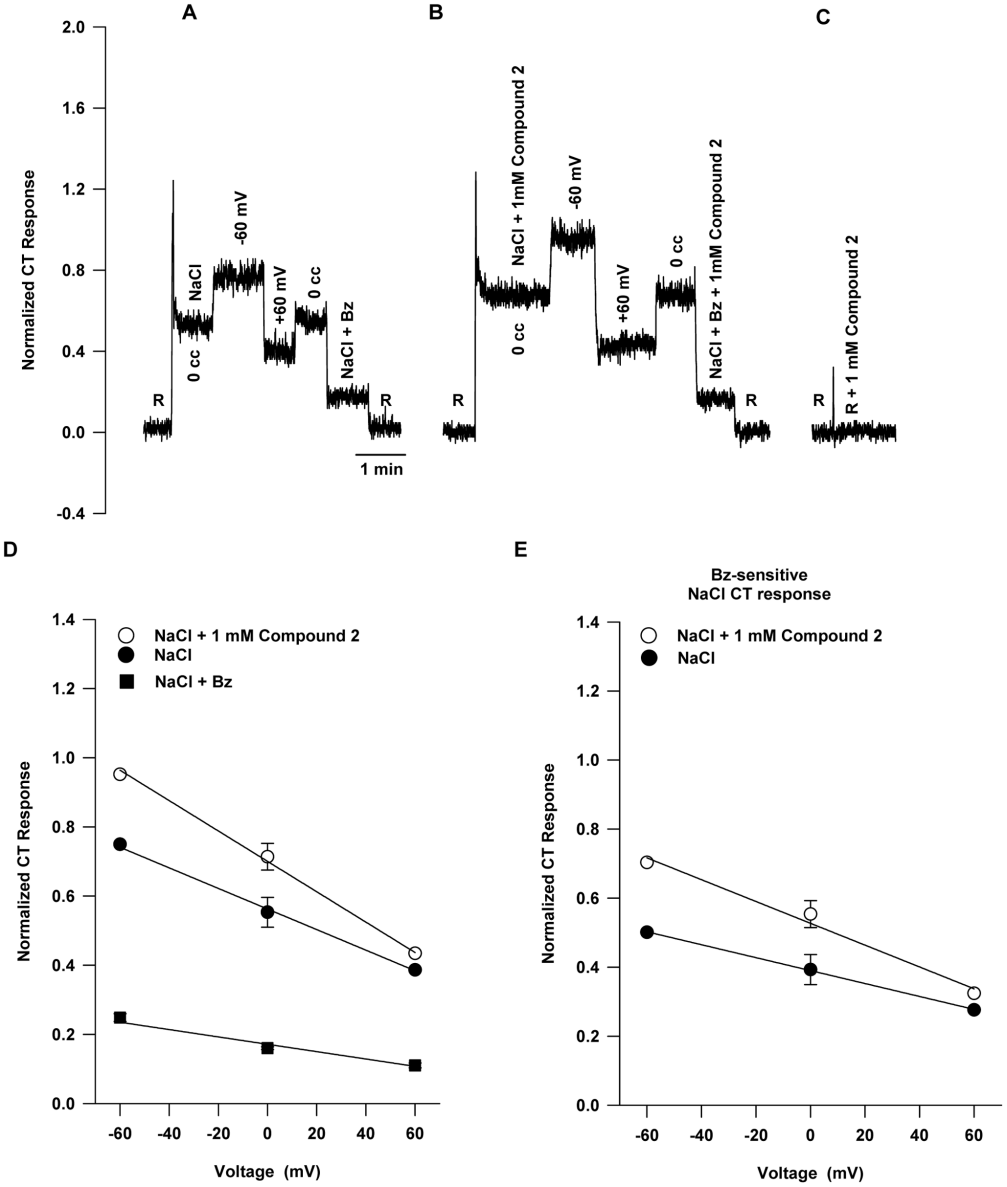
Effect of lingual voltage clamp on rat NaCl CT response in the absence and presence of Compound 2. (**A**) A representative response to 100 mM NaCl under open-circuit (0 cc), and at −60 mV and +60 mV and NaCl+Bz under open-circuit relative to 10 mM KCl rinse (R). (**B**) Response to 100 mM NaCl +1 mM Compound 2 under open-circuit (0 cc), and at −60 mV and +60 mV and NaCl+Bz+Compound 2 under open-circuit. (**C**) 1 mM Compound 2 added to the rinse (R) gave no tonic CT response. (**D**) NaCl+Bz same as in Fig. 2D (*r_o_* = 0.173±0.009; *κ* = 0.0011±0.0002). The NaCl control curve gave in this case the parameter values: *r_o_* = 0.563±0.007; *κ* = 0.0030±0.0001 and NaCl+Compound 2 gave: *r_o_* = 0.700±0.010; *κ* = 0.0043±0.0002. For the NaCl line the increase in *r_o_* represents the presence of ENaC and the increased voltage sensitivity indicates the increase in taste cell membrane conductance due to ENaC. NaCl+Compound 2 line shows the effect of further increasing ENaC conductance (increased *r_o_* and *κ*). (**E**) The Bz-sensitive part of the responses to 100 mM NaCl with and without 1 mM Compound 2. The values are mean ± SEM of 3 rats.

Figure 4 shows the effect of Compound 2 on the response to NaCl under open-circuit, at −60 mV and +60 mV voltage clamp, and under open-circuit with added Bz relative to 10 mM KCl rinse (R). Figure 4A shows that the control response is comparable to that shown in Fig. 2A. The effect of 1 mM Compound 2 is seen in Fig. 4B. Similar to the Compound 1 effect, relative to control, responses under open-circuit were enhanced along with the difference in responses at −60 mV and +60 mV (see also Fig. 4D). Like Compound 1, Compound 2 had no effect on the open-circuit response to NaCl+Bz. This is consistent with Fig. 3B, i.e. Compound 2 affects only the Bz-sensitive part of the response. Fig. 4C shows that 1 mM Compound 2 dissolved in the rinse solution produced no tonic CT response of its own. Figure 4D shows that Compound 2 enhanced the open-circuit response and the voltage sensitivity similarly to Compound 1 (cf. Fig. 2D). The NaCl+Bz line is the same as in Fig. 2D . Figure 4E illustrates the increase in *r_o_* and *κ* due to Compound 2 on the Bz-sensitive part of the response. The values of the parameters *κ* and *r_o_* for the Bz-sensitive response line without and with Compound 2 are also shown in Table 3.

**Figure 5:**
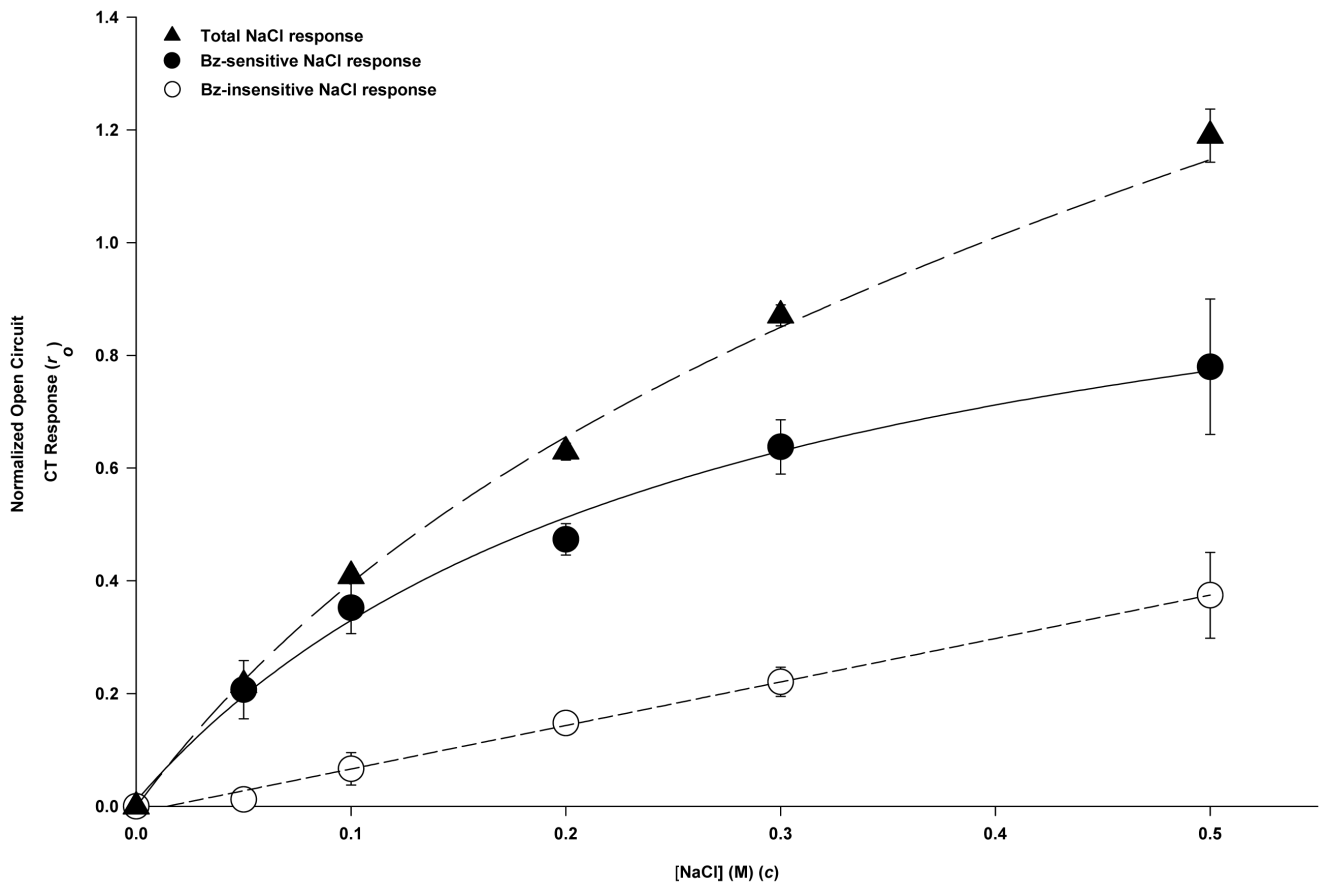
NaCl and NaCl+Bz dose-response curves. Open-circuit normalized CT responses to 50, 100, 200, 300 and 500 mM NaCl in the absence and presence of 5 µM Bz. The Bz-sensitive component was obtained as the difference between the CT response in the absence and presence of Bz. The values are mean ± SEM of 3 rats. The curve for the CT response to NaCl+Bz (Bz-insensitive curve) as a function of the NaCl concentration was linear up to 500 mM NaCl and was accordingly fitted to a regression line (R = 0.998) with intercept = −0.011±0.006 and slope = 0.772±0.023. The curve for the Bz-sensitive response was fitted to eq. 3 which describes the saturation of the open-circuit response, *r_o_*, with increasing NaCl concentration (*r_m_* = 1.20±0.11 and *K_m_* = 0.268±0.061 M).

Figure 5 shows the NaCl concentration versus the CT response curves corresponding to the Bz-sensitive and Bz-insensitive parts of the NaCl CT response along with the total NaCl CT response. The Bz-sensitive part of the response was obtained as the difference of the response to NaCl and the response to NaCl+Bz. The curve for the CT response to NaCl+Bz (Bz-insensitive curve) as a function of the NaCl concentration in Fig. 5 was linear up to 500 mM NaCl and was accordingly fitted to a regression line. The curve for the Bz-sensitive response was fitted to eq. 3 (displayed below) which describes the saturation of the open-circuit response, *r_o_*, with increasing NaCl concentration. The curve fitting the total NaCl CT response was obtained as the sum of the regression line for the Bz-insensitive part of the response and the saturating curve for the Bz-sensitive part of the response. The open-circuit Bz-sensitive NaCl response (*r_o_*) curve (Fig. 5; •) was used to estimate the relative concentration of NaCl that gives an equivalent open-circuit Bz-sensitive NaCl CT response when the rat tongue was stimulated with 100 mM NaCl+Compound 1 (Table 4). The results show that *r_o_* in the presence of 100 mM NaCl +0.5 mM Compound 1 and 100 mM NaCl +1 mM Compound 1 (Fig. 1B) was equivalent to the response observed in the presence of 200 and 300 mM NaCl alone, respectively (Table 4).

**Table 4:** Relative concentration of NaCl that gives an equivalent open-circuit Bz-sensitive NaCl CT response (*r_o_*) in the absence and presence of ENaC modulators.

NaCl (mM)	a *r_o_*	100 NaCl+Compound 1 (mM)	b *r_o_*	BAPTA-AM	*^c^r_o_*	pH	*^d^r_o_*	8-CPT-cAMP	*^e^r_o_*
100	0.33±0.05	0.0	0.37±0.03	Control	0.37±0.03	7.0	0.37±0.03	Control	0.37±0.03
200	0.51±0.03	0.5	0.49±0.02						
300	0.63±0.05	1.0	0.66±0.05	Post-	0.70±0.01	10.3	0.61±0.03	Post-	0.63±0.04

Control = Mean control value from Table 3.

a ***r_o_*** values are from Fig. 5B.

b ***r_o_,*** ^c^ ***r_o_*** **,** ^d^ ***r_o_***, and ^e^ ***r_o_*** values are from Table 3.

b ***r_o_*** value at 200 mM NaCl is from Fig. 1B.

### Effect of 8-CPT-cAMP on the NaCl CT Response

**Figure 6:**
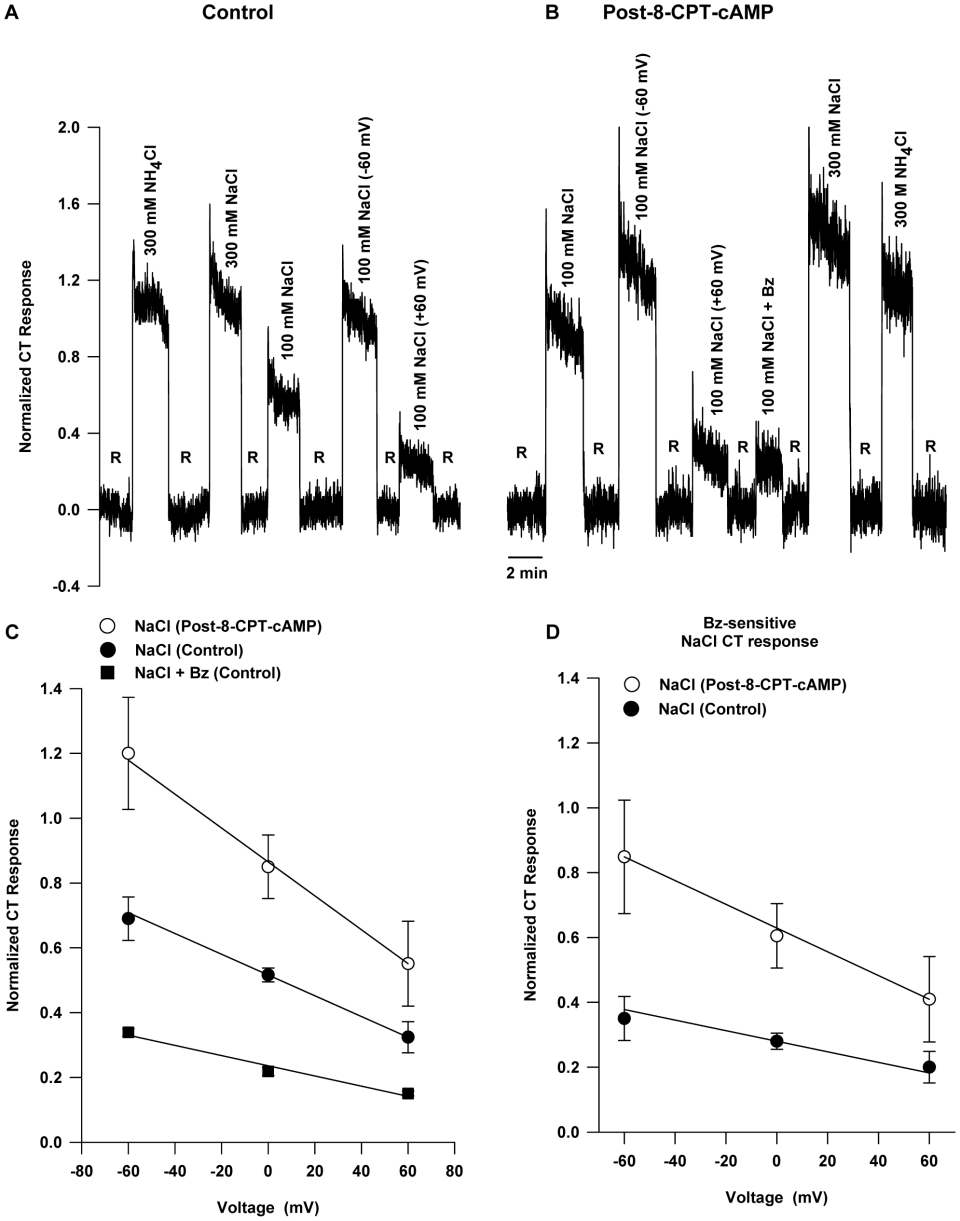
Effect of lingual voltage clamp on rat NaCl CT response before and after topical lingual application of 8-CPT-cAMP. (**A**) Control responses to 300 mM NH_4_Cl, 300 mM NaCl, 100 mM NaCl (open-circuit; 0 cc), 100 mM NaCl (−60 mV) and 100 mM NaCl (+60 mV) relative to 10 mM KCl rinse (R). (**B**) Responses following exposure of rat tongue to 20 mM 8-CPT-cAMP for 30 min. Responses to 100 mM and 300 mM NaCl are enhanced, but the response to 300 mM NH_4_Cl is unaffected. (**C**) The NaCl+Bz and NaCl lines give *r_o_* and *κ* values characteristic of the Bz-insensitive response and total response respectively. For the Bz-insensitive response line (NaCl+Bz) the parameter values were: *r_o_* = 0.236±0.012 and *κ* = 0.0016±0.0002, for the control NaCl response *r_o_* = 0.516±0.006 and *κ* = 0.0032±0.0001, and for the response post-8-CPT-cAMP treatment *r_o_* = 0.865±0.025 and *κ* = 0.0052±0.0005. (**D**) Bz-sensitive NaCl CT response versus voltage under control conditions and post-8-CPT-cAMP exposure. The values are mean ± SEM of 3 rats.

Arginine vasopressin and cAMP have been shown to increase the amiloride-sensitive Na^+^ current in isolated taste bud cells [25] and the NaCl CT response in rats [14]. Figure 6A shows the control response to NaCl under open-circuit conditions and at applied clamp voltages of either –60 mV or +60 mV. Fig. 6B shows similar responses after topical lingual application of 8-CPT-cAMP. The CT response to NaCl at open-circuit and the CT responses under lingual voltage clamp were greater after 8-CPT-cAMP exposure relative to control. 8-CPT-cAMP also produced an increase in the CT response to 300 mM NaCl. No change was observed in the CT response to 300 mM NH_4_Cl after 8-CPT-cAMP treatment. As shown in Table 4, the mean control open-circuit Bz-sensitive NaCl CT response at 100 mM NaCl (*r_o_*) increased from 0.37±0.03 to 0.63±0.04 post-8-CPT-cAMP and was equivalent to the response observed in the presence of 300 mM NaCl alone.

The enhanced response due to 8-CPT-cAMP also varied linearly with voltage (Fig. 6C). Figure 6D displays the variation of the Bz-sensitive part of the response with voltage for the control and post-8-CPT-cAMP cases. The values of the parameter set *κ* and *r_o_* for the Bz-sensitive response line, obtained from the least squares fit of the data, before and post-8-CPT-cAMP are also summarized in Table 3.

Topical application of an equivalent concentration of 8-CPT-cGMP did not produce changes in the NaCl CT response relative to control (data not shown). This suggests that these effects are specific for 8-CPT-cAMP. The 8-CPT-cAMP induced increase in the Bz-sensitive NaCl CT response was not observed when the rat tongue was treated with Rp-8-CPT-cAMP (Figure S1). These results suggest that cAMP-induced enhancement in the NaCl CT response is protein kinase A (PKA) dependent. Increasing the endogenous cAMP levels in fungiform taste bud cells by treating the tongue with IBMX+forskolin enhanced the CT response to both 300 mM NaCl and 100 mM NaCl (Figure S2). Note again that IBMX+forskolin treatment did not alter the CT response to 300 mM NH_4_Cl.

### Effect of [Ca^2+^]_i_ and pH_i_ on the Bz-sensitive NaCl CT Response

Similar results for BAPTA-AM, pH_i_ and ionomycin+Ca^2+^ are shown respectively in Figures S3A, S3B and S3C. Figure S3A shows that decreasing [Ca^2+^]_i_ in the taste cell compartment in which chemosensory transduction occurs [12] caused an increase of in *κ* and *r_o_* in the post-BAPTA-AM Bz-sensitive response versus voltage line relative to control. Figure S3B shows that increasing pH_i_, in the nominal absence of external Ca^2+^ had a comparable effect on the Bz-sensitive response. The values of the parameter set *κ* and *r_o_* for the Bz-sensitive response lines, obtained from the least squares fit of the data, pre- and post-BAPTA-AM and at pH_o_ 7.0 and 10.3 are also summarized in Table 3. As shown in Table 4, the value of *r_o_* after BAPTA-AM treatment (0.70±0.01) or at pH 10.3 (0.61±0.03) was equivalent to the Bz-sensitive NaCl CT response observed in the presence of 300 mM NaCl alone. Figure S3C shows that increasing taste cell [Ca^2+^]_i_ produced a pronounced suppression in the Bz-sensitive NaCl response at all lingual voltages tested. The values of the parameter set *κ* and *r_o_* for the Bz-sensitive response line, obtained from the least squares fit of the data, pre- and post-ionomycin+Ca^2+^ are also summarized in Table 3. Under these conditions the Bz-sensitive component is inhibited to the rinse baseline value.

### Effect of 8-CPT-cAMP and Ionomycin+Ca^2+^ on the Unilateral Apical Na^+^ Influx in Polarized Fungiform Taste Bud Cells

Treating the basolateral membrane of polarized fungiform taste bud cells with 150 µM 8-CPT-cAMP increased the F_490_ fluorescence, and thus the unilateral apical Na^+^ influx into taste bud cells (Figure S4). We have previously shown that increasing taste cell [Ca^2+^]_i_ specifically inhibits the Bz-sensitive NaCl CT response without altering the Bz-insensitive component [12]. To test if 8-CPT-cAMP induced increase in unilateral apical Na^+^ influx is also blocked by increasing taste cell [Ca^2+^]_i_, at the end of the 8-CPT-cAMP experiment, the basolateral compartment was perfused with 0 Na^+^-Ringer’s containing 3 µM ionomycin for 5 min. Following ionomycin treatment, the unilateral apical Na^+^ influx was inhibited below its control value (Figure S4). These results suggest that an increase in [Ca^2+^]_i_ inhibits both the constitutive ENaC activity as well as the 8-CPT-cAMP-induced increase in ENaC activity in fungiform taste bud cells.

### Mechanism of Agonist Action on ENaC

**Figure 7:**
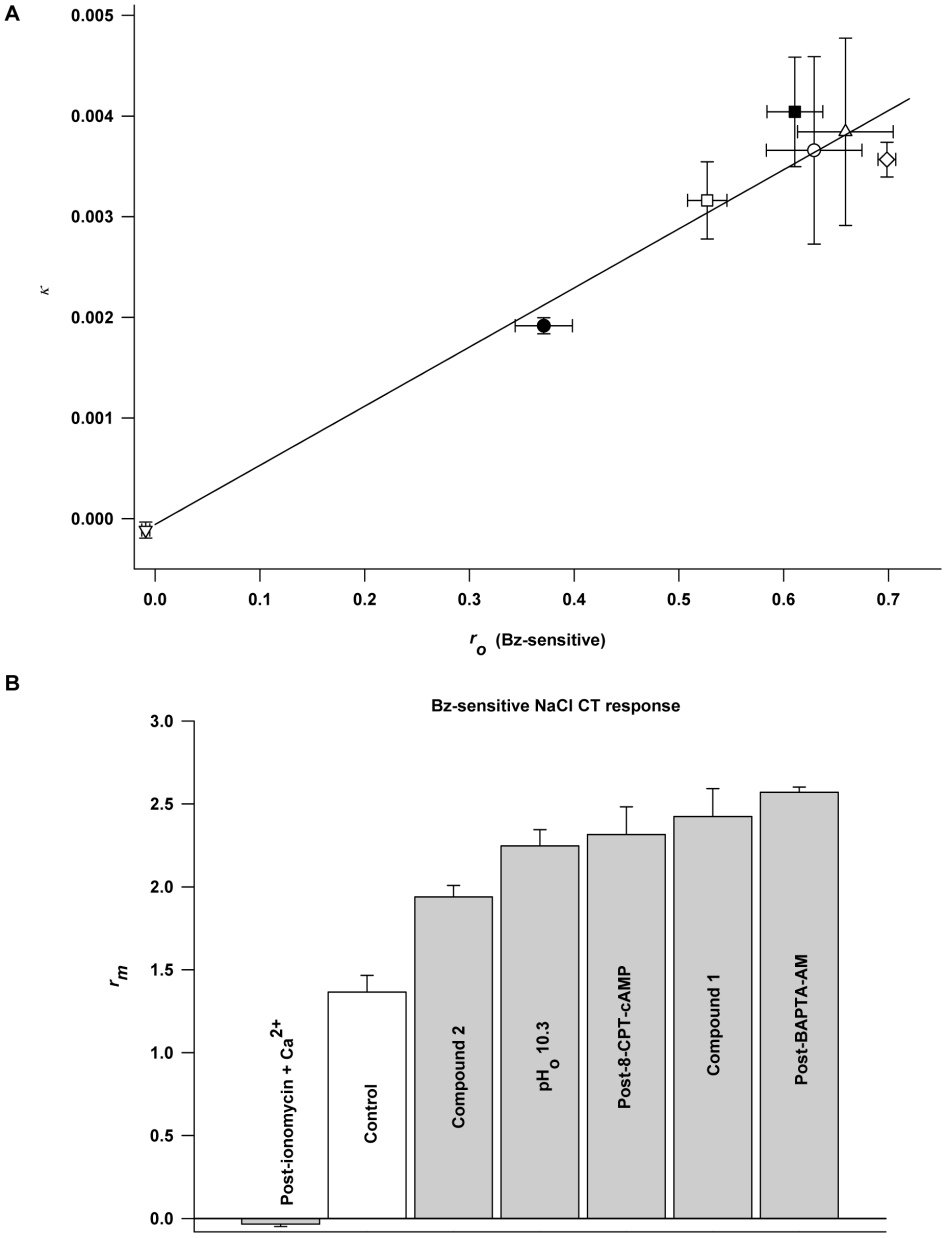
Voltage sensitivity, *κ*, is a linear function of the Bz-sensitive part of the open-circuit response, *r_o_*. (**A**) Indicates that none of the agonists tested exert their effect on the response by changing the kinetic rate constants of the *K_m_* parameter (see text). Agonist Key: (•) control (no agonist), (□) 1 mM Compound 2, (▪) 20 mM 8-CPT-cAMP, (○) pH_o_ 10.3, (▵) 1 mM Compound 1, (⋄) 33 mM BAPTA-AM, (∇) 150 µM ionomycin +10 mM Ca^2+^. R = 0.98. Slope of regression line: 0.0059±0.0005, intercept: −0.57×10^−4^±3.0×10^−4^. (**B**) The maximum Bz-sensitive NaCl CT response (*r_m_*) is the Na^+^ channel parameter that is regulated by the agonists investigated. Relative to the mean *r_m_* for the NaCl control response (no agonist) the mean *r_m_* of all trials with agonist were either statistically larger or smaller. *P* values for larger *r_m_* are as follows: Compound 2 (*P* = 0.0072), pH 10.3 (*P* = 0.0012), cAMP (*P* = 0.0021), Compound 1 (*P* = 0.0012), BAPTA (*P* = 0.0001). *P* value for smaller *r_m_*: ionomycin+Ca^2+^ (*P*<0.0001).

Each ENaC agonist investigated caused *r_o_* and *κ* to increase relative to control (cf. Figs. 2E, 4E, 6D, Figures S3A and S3B). In contrast, with ionomycin+Ca^2+^ treatment, both *r_o_* and *κ* decreased relative to control (Figure S3C). Figure 7A shows the plot of mean *κ* as a function of mean *r_o_* for the control state, for each of the agonists, and for ionomycin+Ca^2+^. It is noted that all the points fall on a straight line that, not surprisingly, passes through the origin. This is consistent with the idea that if ENaC is in a state of zero conductance (*κ* = 0), then it must yield zero response (*r_o_* = 0). It should be noted that the antagonistic condition (ionomycin+Ca^2+^) falls essentially at the origin (cf. Figure S3C), that control conditions fall on the point with coordinates: mean *r_o_* = 0.371±0.067, and mean *κ* = 0.0019±0.0001. The points corresponding to BAPTA-AM, 8-CPT-cAMP, pH 10.3, and Compound 1 all cluster about the mean coordinates: *r_o_* = 0.649±0.070, *κ* = 0.0038±0.0014. This suggests that the Bz-sensitive response to 100 mM NaCl has a natural maximum which is achieved with 33 mM BAPTA, 20 mM 8-CPT-cAMP, 1 mM Compound 1, and pH 10.3. The point corresponding to Compound 2, which has not yet achieved maximum response enhancement at 1 mM (cf. Fig. 3B), falls just outside the maximum cluster. It would appear, therefore, that the intact functioning taste sensory system can produce an enhanced Bz-sensitive response to 100 mM NaCl only up to a maximum limit of about 0.649±0.070, irrespective of the agonist employed, or about a 75% over control.

The fact that *r_o_* and *κ* are related linearly (Fig. 7A) places constraints on the mechanism of agonist enhancement of ENaC activity, and this is explored below. The Bz-sensitive CT response, *r*, is a function of stimulus NaCl concentration, *c*, and the potential difference, *V*, across the anterior tongue under stimulation [15]. For convenience, it is useful to define a dimensionless potential difference *φ*, equal to *δFV/RT* where *δ* is the fraction of *V* dropped across the apical membrane containing ENaC, and the thermodynamic constants *RT*/*F* ( = 26 mV) provide the usual potential scaling. We then have:
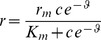
(2)and with *φ = *0, we obtain the open-circuit response, *r_o_*

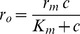
(3)where *r_m_* is the maximum response and *K_m_* is the NaCl concentration at which *r_o_* is half maximal. Since *r* has been shown in each case to be linear with respect to *V* between −60 and +60 mV, we proceed by linearizing the term in *φ* in eq. 2 which yields:



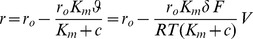
(4)Consistent with Figs. 2E, 4E, 6D, and Figures S3A, S3B, S3C, the model predicts that *r* will vary linearly with *V* with intercept *r_o_* and slope -*κ*. Moreover, it indicates that *κ* and *r_o_* should be proportional, viz
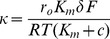
(5)which is consistent with the data in Fig. 7A. For that proportionality to hold the slope in Fig. 7A should be: *K_m_δF*/(*RT*(*K_m_*+*c*)) and have the constant value 0.0059. This means that *K_m_* and *δ* do not vary with the state of the channel, i.e. between mean control coordinates in Fig. 7A (0.371, 0.0019) and mean agonist coordinates (0.649, 0.0038) or antagonist coordinates (−0.009, −0.0001) *K_m_* and *δ* do not vary. Since *K_m_* for the Bz-sensitive response is known from this (see Fig. 5) and previous studies (*K_m_* = 268 mM) [26], the fraction of the potential dropped across the apical membranes containing ENaC, *δ*, is 0.211, consistent with previous determinations [15].

Since *K_m_* is invariant, the increase in *r* due to an agonist (or decrease due to an antagonist) must result from changes in *r_m_*. From eq. 3, *r_m_* was computed for each of the cases studied here and presented in Fig. 7B. Each agonist condition has a significantly larger *r_m_* value than control and the antagonistic case due to increase in [Ca^2+^]_i_ has a significantly smaller *r_m_* value. Again assuming that the CT response is proportional to the Na^+^ flux through ENaC, the maximum response should be proportional to the maximum Na^+^ flux, *j_m_*. From kinetic analysis of the channel *j_m_* = *Nk_c_* where *N* is the apical membrane density of ENaC and *k_c_* is the Na^+^ dissociation rate constant between the cytosolic side of ENaC and the cytoplasm [15]. Assuming that short time agonist exposure does not change channel density, then each agonist enhances the CT response by increasing *k_c_*. From Fig. 7B the limit on *k_c_* increase would appear to be about 75% above control. The kinetic model also gives some insight on how *k_c_* can increase while *K_m_* remains invariant [15]. From the model:
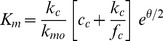
(6)


Here, *k_c_* is the Na^+^ dissociation rate constant between the channel cytosolic side and the cytoplasm, *k_mo_* is the Na^+^ dissociation rate constant between the channel mucosal side and the stimulus solution at zero apical membrane potential, *c_c_* is the cytoplasm Na^+^ concentration, *f_c_* is the Na^+^ association rate constant between the cytoplasm and the channel, and *θ* is the apical membrane potential normalized by *RT/F*. For *K_m_* in eq. 6 to remain constant while *k_c_* increases, it would appear that *θ* must compensate by hyperpolarizing. The extent to which that can occur while the cell retains excitability is probably itself constrained. This would account for the apparent limit in the possible increase in *k_c_* to a maximum of only about 75%.

## Discussion

Investigating the effect of ENaC enhancers on neural and behavioral responses to NaCl is important in identifying salt taste enhancers that may be useful in lowering salt intake. Compound 2 has been identified as a potent small molecule activator of ENaC. At a concentration of 1 µM and 30 µM Compound 2 enhanced the αβγ and δβγ hENaC expressed in oocytes by 300% and 700%, respectively. In contrast, only weak αβγ mENaC activation was observed at 100–300 µM concentrations of Compound 2 [7]. Here we tested the effects of Compound 2 and a structurally related compound (Compound 1) on rat Bz-sensitive NaCl CT responses under open-circuit and voltage clamp conditions. The results presented here address the following important questions related to these small molecule activators of ENaC: (i) Do Compounds 1 and 2 enhance the Bz-sensitive NaCl CT response? (ii) What is the threshold concentration at which these compounds activate the CT response? (iii) What is the maximum enhancement? (iv) What is the concentration of the compound that gives 50% of the maximum response? (v) How do the effects of Compounds 1 and 2 compare with the known physiological activators of ENaC? (iv) What is the underlying mechanism by which ENaC activators enhance the NaCl CT response? Below we discuss how our results provide answers to the above questions.

### Correlation between ENaC Activity and NaCl Neural Response

In rats, typically about 70% of the NaCl CT response is Bz-sensitive (Figs. 1A and 3A). It is expected that modulating the activity of ENaC in fungiform taste cells should have significant effects on neural responses in rats. Consistent with this, Compound 1 and Compound 2 enhanced the Bz-sensitive part of the NaCl CT response (Figs. 1 and 3) without altering the Bz-insensitive NaCl CT response. Compound 1 was effective at lower concentrations (Fig. 1B) than Compound 2 (Fig. 3B). The response threshold for Compound 1 was just above 250 µM, the half-maximal response was at 0.49 mM, and the asymptotic maximal enhanced response was 70% above baseline (Fig. 1B). In contrast, the concentration-response curve for Compound 2 did not reach saturation between 0 and 1 mM and the response threshold was estimated at just above 0.5 mM. Assuming the same sigmoidal-saturation model the half-maximal response was at 1.05 mM. The estimated maximal enhanced response was 87% above baseline (Fig. 3B). The large Hill coefficients for Compound 1 (5.2) and Compound 2 (5.0) suggest significant positive co-operativity for each agonist. While Compound 2 clearly enhances responses mediated by both rENaC and hENaC, and in both cases the concentration-response data fit the same sigmoidal-saturation model, the fit parameters are very different. For hENaC expressed in frog oocytes threshold for Compound 2 was about 30 nM, half-maximal response occurred at 1.2 µM, maximal response was about a 700% above baseline, and the Hill coefficient was 1.0 [7]. This difference must reflect not only the inherently different properties of rENaC and hENaC respectively, but also the very different physiological conditions under which each Na^+^ transducer was studied.

Both Compound 1 and Compound 2 increased the slope of the line representing the response as a function of voltage as well as the open-circuit response (*r_o_*). The negative of the slope is defined as the voltage sensitivity of the curve (*κ*) and as eq. 4 shows it can be considered a kind of global conductance, assuming that the Bz-sensitive response is proportional to the aggregate Na^+^ current through ENaC. From eq. 5 we note that the voltage sensitivity,*κ* (analogous to conductance) to be proportional to the open-circuit response, *r_o_*, and, this is borne out by Fig. 7A. So each of the agonists examined, have in common a measurable increase in *κ* resulting in a proportional increase in *r_o_*. In the presence of ionomycin+Ca^2+^, *κ* and, therefore, *r_o_* were reduced to zero (Fig. 7A and Figure S3C). The fact that the relationship between *κ* and *r_o_* is linear informs us that *K_m_* in the response equations (eq. 2 and 3) remains constant irrespective of the state of ENaC. This means that agonist or antagonist modulation of *κ* and *r_o_* must occur by modulation of the maximum response, *r_m_* alone. From a kinetic model of ENaC [15] this implies that rate constant most affected by agonist action is *k_c_*, the rate constant for the dissociation of Na^+^ from the cytoplasmic side of ENaC and into the cytosol. However, as eq. 6 shows, *k_c_* also appears in the expression for *K_m_*. Since *K_m_* remains constant, we must conclude that any increase in *K_m_* that might result from an increase in *k_c_* is fully compensated by an increased hyperpolarization (*θ*) at the apical cell membrane. This cannot occur without limit, and such a limit may explain, at least in part, why the maximum enhancement in the rENaC activated CT response observed in the intact sensory system with a variety of enhancers amounts to only 75% compared to 700% observed with Compound 2 using hENaC expressed in frog oocytes [7].

Compound 2 seems to exert its agonist action by binding directly to the β subunit of hENaC [7]. On that basis, it seems reasonable to assume that Compound 2 and the structurally related Compound 1 activate an agonist binding site on rENaC in taste cells. ENaC expressed in mouse kidney cortical collecting duct cells has both H^+^ and Ca^2+^ binding sites [27], and we assume similar sites are available on rENaC in taste bud cells. In inside out patch clamp recordings on mouse kidney cortical collecting duct cells, an increase in [Ca^2+^]_i_ produced a significant decrease in ENaC open probability without altering channel conductance. The inhibitory effect was due to a direct interaction between Ca^2+^ and ENaC, and was dependent on [Ca^2+^]_i_ [27]. Changes in pH_i_ also directly regulated ENaC open probability. Lower pH_i_ (<pH 7) reduced the ENaC open probability as shown in shorter opening time, and higher pH_i_ (>pH 7) enhanced the ENaC open probability as shown in augmented opening time without causing any alteration in channel conductance. The effects of pH_i_ on ENaC open probability could be summarized as an S-shaped curve around pH 7.2 [27].

Both an exogenous (Fig. 6) and endogenous (Figure S2) increase in cAMP enhanced the Bz-sensitive NaCl CT response under open-circuit conditions that was PKA dependent (Figure S1). 8-CPT-cAMP increased amiloride-sensitive current by more than 300% in Xenopus oocytes expressing chimeric ENaC channels composed of guinea pig α subunit in combination with rat βγ subunits. However, 8-CPT-cAMP had no effect on wild-type, nonchimeric rENaC channels [28]. This suggests that, unlike rat ENaC, tonic PKA activity is required for basal function of guinea pig α-containing ENaC and that PKA mediates its cAMP-induced activation. Thus, PKA sensitivity of ENaC can depend on the nature of the ENaC α-subunit and raises the possibility that cAMP can stimulate ENaCs by different mechanisms [29]. Vasopressin stimulates the expression/activity of ENaC through the cAMP/PKA pathway in the cortical collecting tubule and may involve additional translocation of channel proteins into the apical cell membrane [23], [30], [31].

The Bz-sensitive component of the NaCl CT response is enhanced by systemic administration of aldosterone [32] and is thought to be due to translocation of ENaC from intracellular locations to the apical membrane in rat taste bud cells [2]. In mice angiotensin II type 1 receptors (AT1) were co-expressed in taste cells containing α-ENaC. Systemic administration of angiotensin II suppressed amiloride-sensitive NaCl CT responses and this effect was blocked by the angiotensin II type 1 receptor (AT1) antagonist CV11974 [33]. It is likely that activation of AT1, a G-protein-coupled receptor, decreases ENaC activity by activating inositol trisphosphate-dependent pathways and an increase in taste cell [Ca^2+^]_i_ [34].

While these sites are likely to be different for each agonist tested, the effect in each case of ENaC conductance on the NaCl CT response seems to be the same, i.e. an increase in *κ* and, therefore, *r_o_* as a result of an increase in the maximum response, *r_m_*, resulting from an increase in *k_c_*, the rate constant for the dissociation of Na^+^ from ENaC to the cytosol. The convergence of each agonist action on the same mechanism of ENaC conductance increase leading to the same degree of enhancement suggests that any new ENaC agonist that also increases *r_m_* and *k_c_* will most likely also demonstrate the same 75% enhancement limit. It is possible, however, that further increase in the enhancement limit may still be achieved by combining the above agonists with mechanisms (e.g. aldosterone administration) that increase the abundance of ENaC channels in the apical membrane of salt sensing taste bud cells [2].

BAPTA-AM and ionomycin+Ca^2+^ alter [Ca^2+^]_i_ in all fungiform taste bud cells. In our previous studies [12], [35], BAPTA-AM inhibited tonic CT responses to acidic and bitter taste stimuli without altering the response to sweet taste stimuli. It significantly enhanced the Bz-sensitive NaCl CT response and produced a small, but significant, increase in the Bz-insensitive NaCl CT response. In contrast, topical lingual application of ionomycin+Ca^2+^ had no effect on the tonic CT response to bitter, sweet and umami taste stimuli [35]. However, it significantly inhibited the tonic CT response to acidic stimuli and the Bz-sensitive NaCl CT response without altering the Bz-insensitive NaCl CT response [35], [36]. Our lingual voltage-clamp data suggest that an increase in taste cell [Ca^2+^]_i_ enhances the Bz-sensitive NaCl CT response by increasing the ENaC-dependent apical membrane conductance in salt sensing taste cells [35]. Thus, our whole nerve recordings, in the absence and presence of BAPTA-AM and ionomycin+Ca^2+^ and using NaCl as the sole stimulus and Bz, as a specific blocker of ENaC, provide taste related information from a specific subset of taste cells involved in salt taste sensing.

No CT response is observed by changing the rinse solution pH from 7.0 to 10.3 [14]. Therefore, the NaCl and NaCl+Bz CT responses reflect changes that arise due to pH effects on the ENaC containing fungiform taste bud cells. Although 8-CPT-cAMP enhanced CT responses to strong acids [37], this effect is not a significant issue here, since experiments were performed at pH_o_ 7.0 and 10.3 that do not elicit a CT response.

Our results further tend to suggest that there may be two sub-compartments, a cytosolic compartment and a synaptic compartment in taste cells in which changes in [Ca^2+^]_i_ play different roles in taste reception. While changes in [Ca^2+^]_i_ in the synaptic compartment in taste cells play a role in neurotransmitter release, changes in [Ca^2+^]_i_ in the cytosolic compartment play a regulatory role in modulating the activity of ion channels, transporters and other downstream intracellular signals in transduction [12], [35]. Both phasic and tonic CT responses are modulated by Bz, ±60 mV applied voltage, and 8-CPT-cAMP (Figs. 6A and 6B) and by modulating taste cell [Ca^2+^]_i_ and pH_i_ [12], it is most likely that both phasic and tonic NaCl CT responses are regulated by similar mechanisms.

### Correlation between ENaC Activity and NaCl Behavior

A specific enhancement in the Bz-sensitive salt taste sensitivity by ENaC enhancers may contribute to lower Na^+^ intake. Alternately, a specific inhibition in the Bz-sensitive salt taste sensitivity by ENaC inhibitors may contribute to increased Na^+^ intake. Inhibiting ENaC activity by amiloride appears to render NaCl qualitatively indistinguishable from KCl [6]. Silencing α-ENaC specifically in mouse taste cells renders them non-responsive to NaCl concentrations that are generally appetitive [3], [4]. In behavioral tests, CV11974, an AT1 antagonist, reduced the stimulated high licking rate to NaCl in water restricted mice. This suggests that a specific reduction of the amiloride-sensitive salt taste sensitivity by angiotensin II may contribute to increased Na^+^ intake [33].

In contrast to the studies in rodents [3], [6], no significant effect of amiloride was observed on perceived salt intensity in human subjects even when tested at levels at approximately 300-fold above the IC_50_ for αβγ ENaC expressed in oocytes and equivalent to approximately 10-fold over the IC_50_ value for δβγ ENaC expressed in oocytes [8], [9]. Since δ ENaC is more than an order of magnitude less sensitive to amiloride than α ENaC [7], and human salt taste is less sensitive to amiloride [38], it is believed that human salt taste may be mediated, in part, by δ ENaC or δβγ ENaC expressed in a subset of human taste bud cells [5]. Although, at present, the effect of Compound 1 and Compound 2 on human salt sensory perception are lacking, ENaC enhancers, in general, have been shown to induce only about 6.2–10.7% change in NaCl concentration detection [39], [40]. One plausible explanation why ENaC modulators do not enhance salt taste in humans may be that δβγ hENaC in taste bud cells operates constitutively at or near its maximum capacity. Therefore, further up-regulating δβγ hENaC activity by channel activators does not alter human salt taste perception to a significant degree. Alternately, these results tend to suggest that δ ENaC and δβγ ENaC may play a minor role in human salt taste transduction.

In summary, our results demonstrate that enhancing ENaC activity by Compound 1, Compound 2, 8-CPT-cAMP, BAPTA-AM and alkaline pH specifically increases the magnitude of the Bz-sensitive NaCl CT response, apical membrane Na^+^ conductance and apical Na^+^ flux across the apical membrane of a subset of fungiform taste bud cells. Unlike the studies on hENaC expressed in oocytes [7], the maximum enhancement (*r_m_*) in the Bz-sensitive NaCl CT response in the intact rat sensory system in the presence of the above ENaC modulators was around 75%.

## Supporting Information

Figure S1 **Effect of Rp-8-CPT-cAMPS on the 8-CPT-cAMP-induced increase in NaCl CT response. (A)** A representative open-circuit CT response to 100 mM NaCl and 100 mM NaCl +5 µM Bz before 8-CPT-cAMP treatment (Control). The open-circuit CT response to 100 mM NaCl and 100 mM NaCl +5 µM Bz is shown in the same rat after 30 min of topical lingual application of 20 mM 8-CPT-cAMP (Post-8-CPT-cAMP) for 30 min. **(B)** A representative response to 100 mM NaCl and 100 mM NaCl +5 µM Bz under open-circuit in another rat under control conditions (Control), after topical lingual application of 4 mM Rp-8-CPT-cAMPS for 20 min (Post-Rp-8-CPT-cAMP), and after 20 mM 8-CPT-cAMP for 30 min (Post-Rp-8-CPT-cAMP-post 8-CPT-cAMP). In 3 such experiments no significant differences were observed in the NaCl or NaCl+Bz tonic CT response under control, Post-Rp-8-CPT-cAMP and Post-Rp-8-CPT-cAMP-post 8-CPT-cAMP conditions (p>0.05, paired).
(TIF)

Figure S2 **Effect of IBMX+froskolin on rat NaCl CT response.** Shows mean normalized tonic NaCl CT responses to 300 mM NH_4_Cl, 300 mM NaCl and 100 mM NaCl before (Control) and after topical lingual application of 100 µM IBMX +100 µM forskolin for 20 min relative to 10 mM KCl rinse (R). The values are mean ± SEM of 4 rats. *p<0.0123 and **p<0.0001 (Paired).
(TIF)

Figure S3 **Effect of lingual voltage clamp on rat NaCl CT response before and after topical lingual application of BAPTA-AM, Ionomycin+Ca^2+^ and alkaline pH.** **(A)** Bz-NaCl CT response versus voltage under control conditions and post-BAPTA-AM exposure. **(B)** Bz-sensitive NaCl CT response versus voltage at pH_o_ 7.0 and pH_o_ 10.3. **(C)** Bz-sensitive NaCl CT response versus voltage under control conditions (10 mM Ca^2+^) and post-ionomycin +10 mM Ca^2+^. In each case the values are mean ± SEM of 3 rats.
(TIF)

Figure S4 **Effect of 8-CPT-cAMP and ionomycin+Ca^2+^ on the unilateral apical Na^+^ flux in polarized fungiform taste bud cells.** Initially, sodium green loaded polarized fungiform taste bud cells were perfused bilaterally with 0 Na^+^-Ringer’s solution. The unilateral apical Na^+^ influx was measure as the maximum increase in F_490_ induced by unilaterally changing the apical 0 Na^+^-Ringer’s solution with 150 mM Na^+^-Ringer’s (Control). Changes in F_490_ were again measured after treating the basolateral membrane of polarized fungiform taste bud cells with 150 µM 8-CPT-cAMP for 10 min (8-CPT-cAMP). Changes in F_490_ were again measured after treating the basolateral membrane of polarized fungiform taste bud cells with 3 µM ionomycin for 10 min (Ionomycin+Ca^2+^). The F_490_ value in each region of interest (ROI) at 150 mM Na^+^-Ringer’s was compared with the F_490_ value at 0 Na^+^-Ringer’s solution, which was taken as 100%. The values are presented as mean ± SEM of 3 polarized fungiform taste bud preparations using 18 ROIs. *p<0.0346; **p<0.0036 (Paired).
(TIF)
