# Supplementary figures and images for: Effect of ENaC Modulators on Rat Neural Responses to NaCl
Source: PLoS One. 2014 May 19;9(5):e98049. doi: 10.1371/journal.pone.0098049 (PMC4026388; doi:10.1371/journal.pone.0098049)

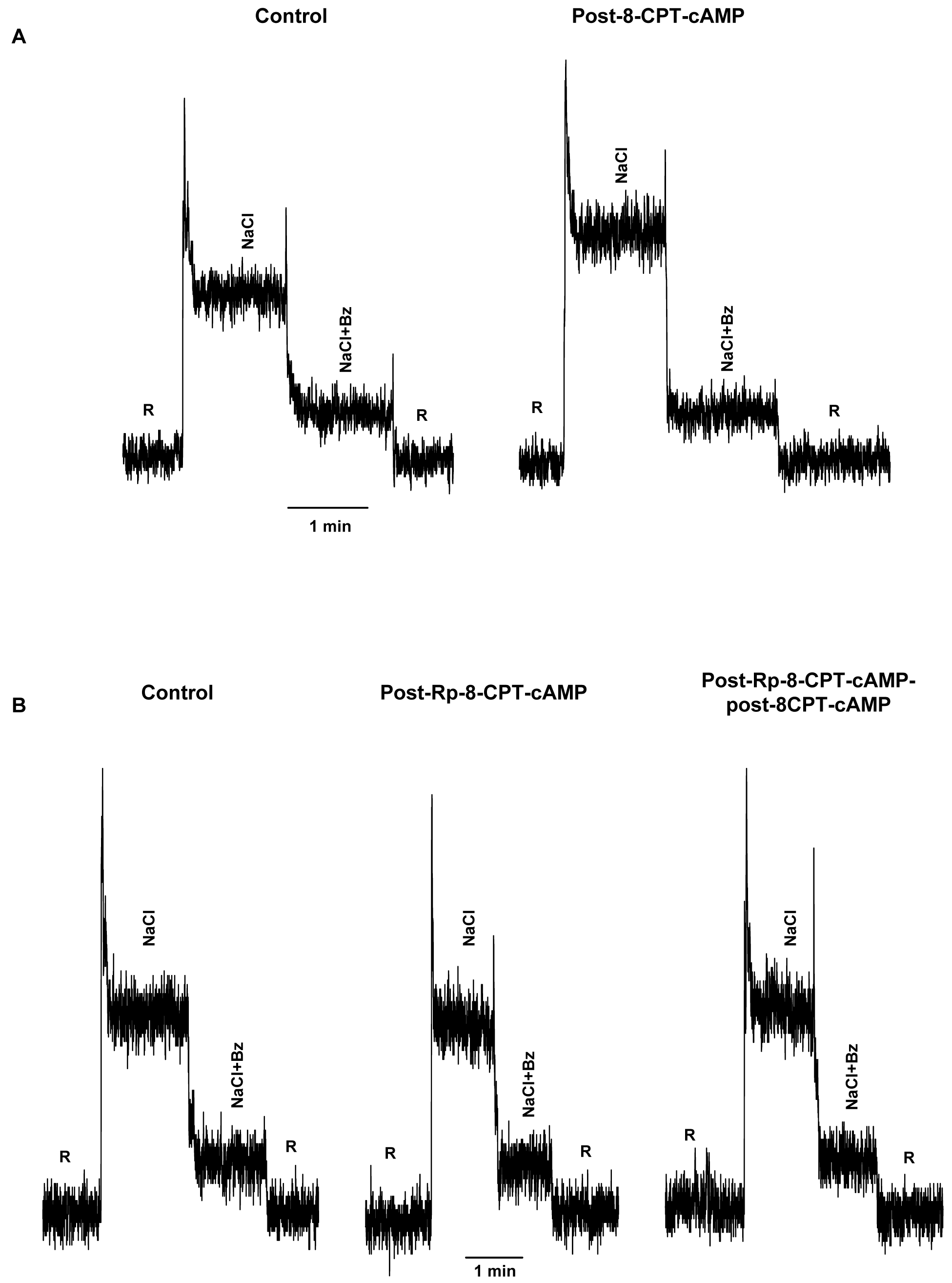

Supplement: Figure S1 — Effect of Rp-8-CPT-cAMPS on the 8-CPT-cAMP-induced increase in NaCl CT response. (A) A representative open-circuit CT response to 100 mM NaCl and 100 mM NaCl +5 µM Bz before 8-CPT-cAMP treatment (Control). The open-circuit CT response to 100 mM NaCl and 100 mM NaCl +5 µM Bz is shown in the same rat after 30 min of topical lingual application of 20 mM 8-CPT-cAMP (Post-8-CPT-cAMP) for 30 min. (B) A representative response to 100 mM NaCl and 100 mM NaCl +5 µM Bz under open-circuit in another rat under control conditions (Control), after topical lingual application of 4 mM Rp-8-CPT-cAMPS for 20 min (Post-Rp-8-CPT-cAMP), and after 20 mM 8-CPT-cAMP for 30 min (Post-Rp-8-CPT-cAMP-post 8-CPT-cAMP). In 3 such experiments no significant differences were observed in the NaCl or NaCl+Bz tonic CT response under control, Post-Rp-8-CPT-cAMP and Post-Rp-8-CPT-cAMP-post 8-CPT-cAMP conditions (p>0.05, paired). (TIF) [file pone.0098049.s001.tif]

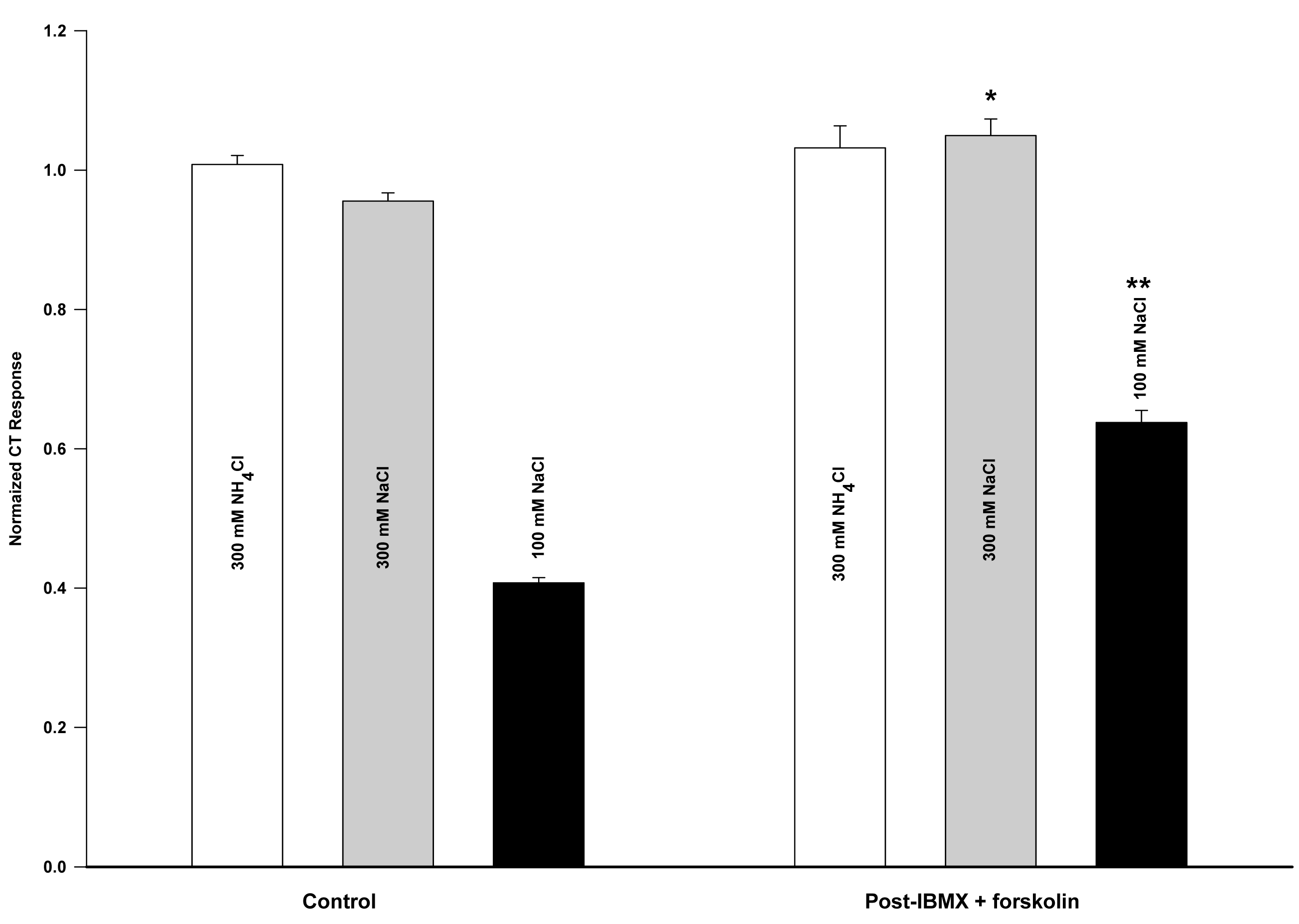

Supplement: Figure S2 — Effect of IBMX+froskolin on rat NaCl CT response. Shows mean normalized tonic NaCl CT responses to 300 mM NH4Cl, 300 mM NaCl and 100 mM NaCl before (Control) and after topical lingual application of 100 µM IBMX +100 µM forskolin for 20 min relative to 10 mM KCl rinse (R). The values are mean ± SEM of 4 rats. *p<0.0123 and **p<0.0001 (Paired). (TIF) [file pone.0098049.s002.tif]

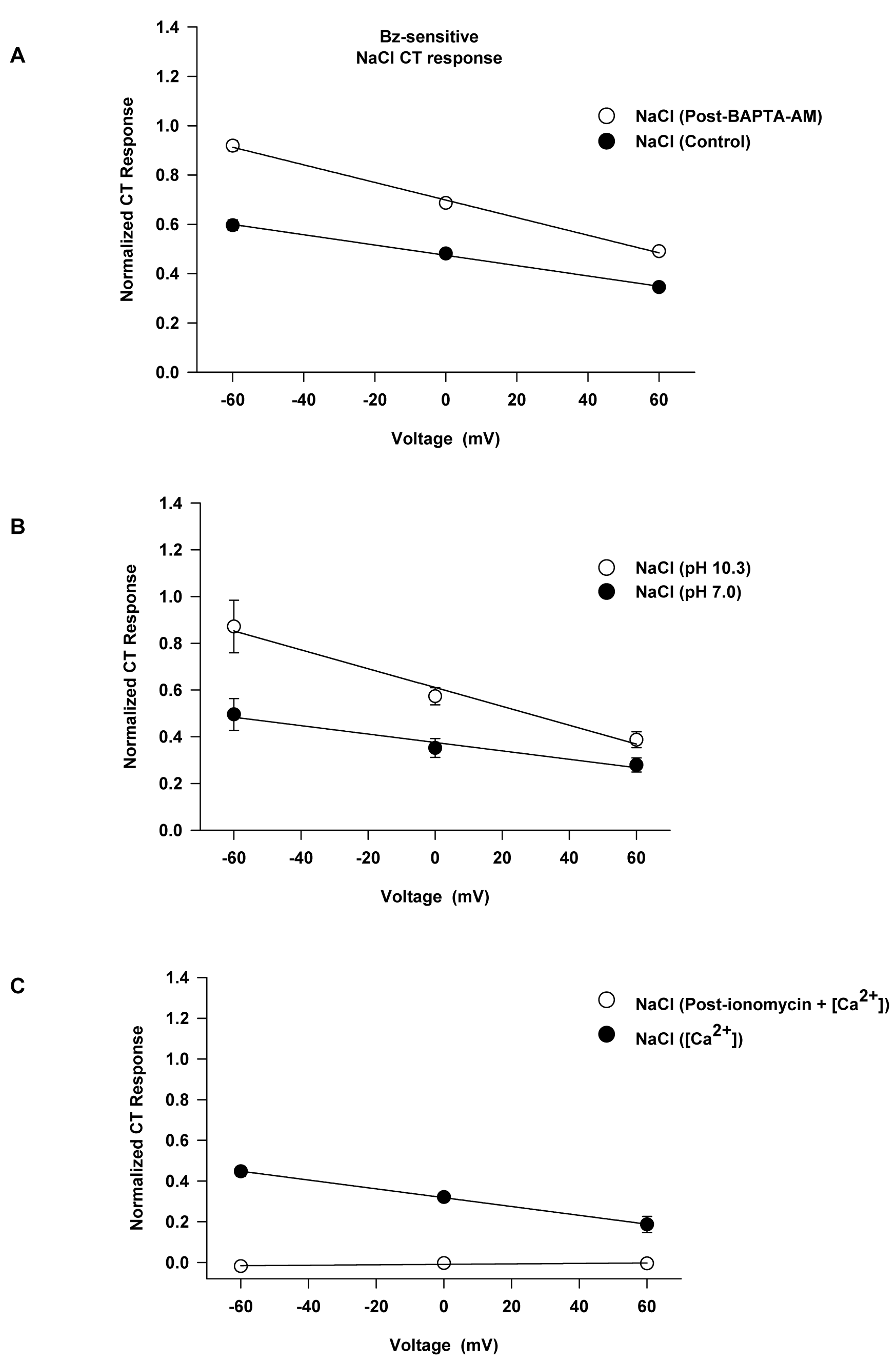

Supplement: Figure S3 — Effect of lingual voltage clamp on rat NaCl CT response before and after topical lingual application of BAPTA-AM, Ionomycin+Ca2+ and alkaline pH. (A) Bz-NaCl CT response versus voltage under control conditions and post-BAPTA-AM exposure. (B) Bz-sensitive NaCl CT response versus voltage at pHo 7.0 and pHo 10.3. (C) Bz-sensitive NaCl CT response versus voltage under control conditions (10 mM Ca2+) and post-ionomycin +10 mM Ca2+. In each case the values are mean ± SEM of 3 rats. (TIF) [file pone.0098049.s003.tif]

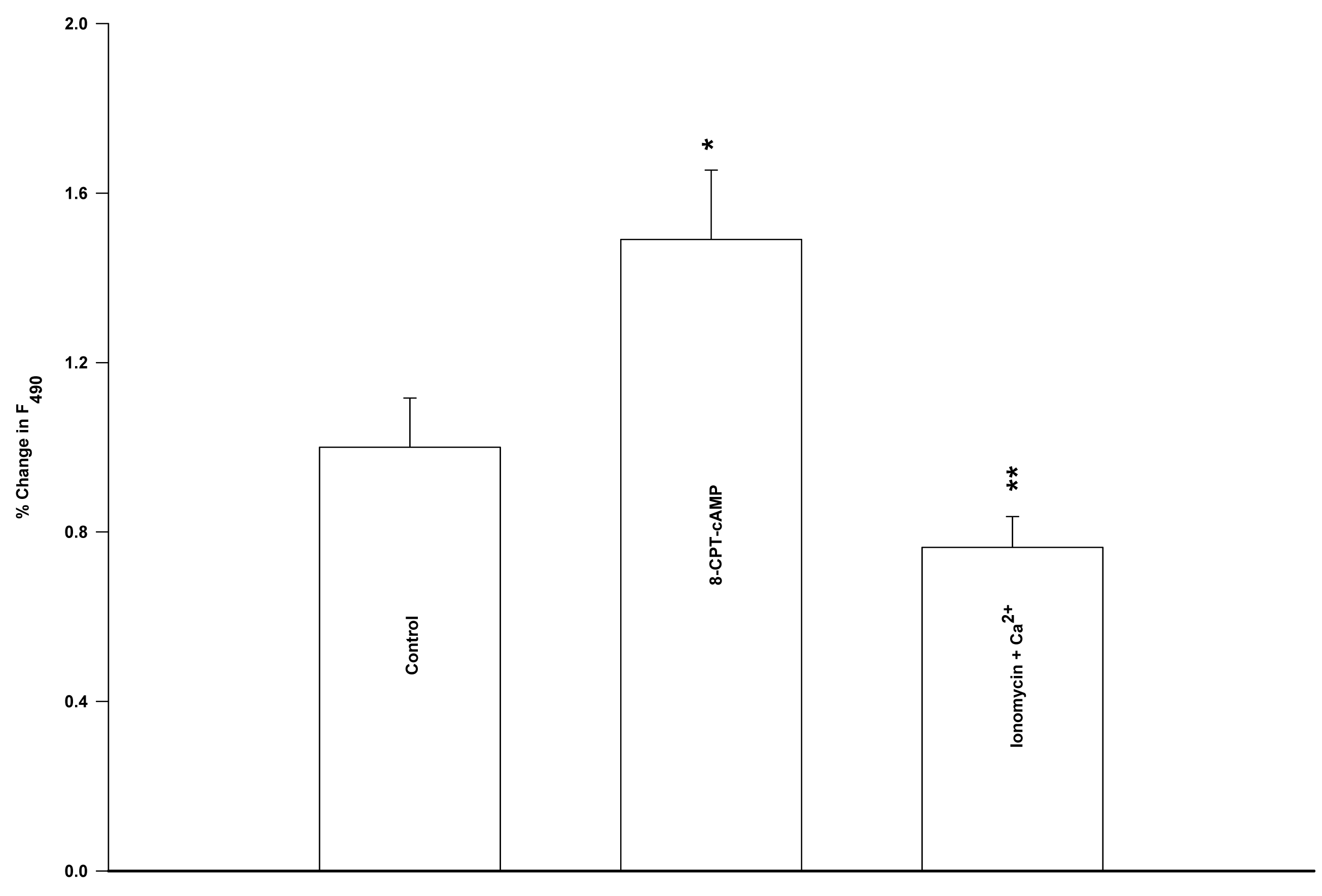

Supplement: Figure S4 — Effect of 8-CPT-cAMP and ionomycin+Ca2+ on the unilateral apical Na+ flux in polarized fungiform taste bud cells. Initially, sodium green loaded polarized fungiform taste bud cells were perfused bilaterally with 0 Na+-Ringer’s solution. The unilateral apical Na+ influx was measure as the maximum increase in F490 induced by unilaterally changing the apical 0 Na+-Ringer’s solution with 150 mM Na+-Ringer’s (Control). Changes in F490 were again measured after treating the basolateral membrane of polarized fungiform taste bud cells with 150 µM 8-CPT-cAMP for 10 min (8-CPT-cAMP). Changes in F490 were again measured after treating the basolateral membrane of polarized fungiform taste bud cells with 3 µM ionomycin for 10 min (Ionomycin+Ca2+). The F490 value in each region of interest (ROI) at 150 mM Na+-Ringer’s was compared with the F490 value at 0 Na+-Ringer’s solution, which was taken as 100%. The values are presented as mean ± SEM of 3 polarized fungiform taste bud preparations using 18 ROIs. *p<0.0346; **p<0.0036 (Paired). (TIF) [file pone.0098049.s004.tif]
